# Phytochemicals and Amino Acids Profiles of Selected sub-Saharan African Medicinal Plants’ Parts Used for Cardiovascular Diseases’ Treatment

**DOI:** 10.3390/pharmaceutics13091367

**Published:** 2021-08-31

**Authors:** Johnson Oluwaseun Odukoya, Julianah Olayemi Odukoya, Edwin Mpoh Mmutlane, Derek Tantoh Ndinteh

**Affiliations:** 1 Centre for Natural Products Research, Department of Chemical Sciences, University of Johannesburg, P.O. Box 17011, Doornfontein, Johannesburg 2028, South Africa; edwinm@uj.ac.za; 2 Department of Chemistry, The Federal University of Technology, Akure PMB 704, Ondo State, Nigeria; 3 Department of Biotechnology and Food Technology, Faculty of Science, University of Johannesburg, P.O. Box 17011, Doornfontein, Johannesburg 2028, South Africa; julianahodukoya@gmail.com; 4 Department of Food Science and Technology, Kwara State University, Malete, Ilorin PMB 1530, Kwara State, Nigeria

**Keywords:** amino acid score, bioactive compounds, branched-chain amino acids, essential amino acids, food safety, FTIR spectroscopy, heart disease, medicinal food plants, principal component analysis, protein quality

## Abstract

For years, the focus on the lipid–atherosclerosis relationship has limited the consideration of the possible contribution of other key dietary components, such as amino acids (AAs), to cardiovascular disease (CVD) development. Notwithstanding, the potential of plant-based diets, some AAs and phytochemicals to reduce CVDs’ risk has been reported. Therefore, in this study, the phytochemical and AA profiles of different medicinal plants’ (MPs) parts used for CVDs’ treatment in sub-Saharan Africa were investigated. Fourier-transform infrared analysis confirmed the presence of hydroxyl, amino and other bioactive compounds’ functional groups in the samples. In most of them, glutamic and aspartic acids were the most abundant AAs, while lysine was the most limiting. *P. biglobosa* leaf, had the richest total branched-chain AAs (BCAAs) level, followed by *A. cepa* bulb. However, *A. cepa* bulb had the highest total AAs content and an encouraging nutraceutical use for adults based on its amino acid score. Principal component analysis revealed no sharp distinction between the AAs composition of MPs that have found food applications and those only used medicinally. Overall, the presence of medicinally important phytochemicals and AAs levels in the selected MPs’ parts support their use for CVDs treatment as they might not add to the AAs (e.g., the BCAAs) burden in the human body.

## 1. Introduction

Scientific reports have revealed that cardiovascular diseases (CVDs)—a group of heart and blood vessels’ disorders involving pathologic process (usually atherosclerosis)—are the number one global cause of death [1,2,3,4,5,6]. These diseases, which include coronary artery disease, coronary heart disease, cerebrovascular disease, rheumatic heart disease, heart failure, stroke [2,3,5,6,7,8] and other conditions with diabetes mellitus, high blood pressure, hyperlipidemia, obesity, physical inactivity, smoking, increased age and family history as some of the risk factors [1,9], claim the lives of approximately 17.9 million people annually [6]. Myocardial infarction (heart attack), one of the manifestations of CVDs [1], occurs after prolonged ischemia (reduced blood flow) of the coronary arteries [10].

As most of the deaths arising from CVDs are in low- and middle-income countries [5], traditional medicine, involving the application of medicinal plants (MPs), has been used for treatment. This can be attributed to the inadequate primary health care systems, limited access to modern health services [11,12,13,14] and unavailability/high cost of modern medicine [12,14,15] in some of these countries. The WHO [16] described herbal medicine to include herbs, herbal materials, herbal preparations and finished herbal products that have parts of plants, other plant materials, or combinations as active ingredients.

Meanwhile, atherosclerosis development has been indicated as the primary cause of CVDs [5] in which cholesterol deposits within the artery; triglycerides and their chief components, fatty acids, are contributing factors. The attention on the lipid–atherosclerosis relationship has, however, led to less consideration of the possible contribution of other key dietary components, such as amino acids (AAs), to atherogenesis and CVDs’ development [5]. Among the >300 of these AAs occurring in nature, 20 serve as building blocks of protein [17]. The essential AAs (EAAs), or indispensable AAs [18], are those whose carbon skeletons cannot be synthesized or are inadequately synthesized de novo by the body based on their needs and must be obtained from the diet to satisfy optimal requirements, while the non-essential AAs (NEAAs) are those that can be synthesized de novo by the body in required sufficient amounts [17]. Functional AAs, which include arginine, cysteine, glutamine, leucine, proline and tryptophan, help in the regulation of important metabolic pathways needed for maintenance, growth, reproduction and immunity [17].

The branched-chain AAs (BCAAs) comprising isoleucine (Ile), leucine (Leu) and valine (Val) are a sub-group of EAAs in humans [19,20,21]. Similar to other EAAs, they are mainly obtained via dietary protein intake [20] with rich levels found in meat, fish, dairy products and eggs [21]. Nevertheless, McDougall [22] pointed out the possibility of the consumption of animal protein contributing to some human health-related problems such as heart disease, diabetes and obesity, among others. Olsen et al. [23] also reported that studies have related a high regular intake of animal protein with increased adiposity, whereas the prevention of CVDs and some of their risk factors has been linked with plant-based diets.

Broadly, BCAAs are important for normal growth/function at the cellular and organ levels [5,24] with vital mediation effects on protein synthesis, glucose homeostasis, anti-obesity as well as nutrient-sensitive signaling pathways [25]. Despite these, their accumulation and that of related metabolites may bring about negative effects [19]. For instance, they have been linked with several cardiometabolic risk factors such as high blood pressure and dyslipidemia [26]. White and Newgard [21] reported that a chronic rise in BCAAs is observed in blood from individuals with obesity-associated conditions such as insulin resistance, type 2 diabetes and CVDs. In addition to BCAAs, Ntzouvani et al. [27] revealed that aromatic AAs (ArAAs), particularly phenylalanine (an EAA) and tyrosine (a NEEA), have also been associated with cardiometabolic risk. Other potential atherogenic AAs that have been indicated include glutamate, glutamine, methionine (with its metabolic intermediate) and homocysteine [5].

On the other hand, phytochemicals, also referred to as phytonutrients [28], are natural bioactive compounds in plants with human health benefits of preventing and reducing the risk of chronic diseases such as CVDs [29,30,31,32,33,34]. According to Geetha et al. [31] and Patle et al. [35], they are classified into two categories, namely, primary and secondary metabolites in which the former include proteins, while their general profiling can be carried out using Fourier-transform infrared (FTIR) spectroscopy. FTIR spectroscopy has been indicated as a fast, versatile, cheap, non-destructive and effective analytical technique for carrying out chemical constituents analysis of biological materials [35,36,37,38], including plant matrices [39]. It aids the detection, characterization and identification of the key functional groups and chemical bonds of bioactive compounds present in the sample [31,38].

As infrared (IR) spectrometry helps in herbal analysis [36], and dietary intake, among other factors, may contribute to the elevation of BCAAs in circulation [19], this research was aimed at assessing the phytochemicals profile and principally the distribution of AAs (including BCAAs and ArAAs) in eight MPs’ parts used for the treatment of CVDs as well as their associated risk factors in sub-Saharan Africa (SSA). This becomes necessary as (1) plants contain phytochemicals in addition to essential nutrients [29]; (2) the association of several AAs such as BCAAs, phenylalanine, tyrosine, glutamate, glutamine, methionine and homocysteine with atherosclerosis development, CVDs risk and related cardio-metabolic disorders has been reported [5,19,21,26]; (3) different plants’ parts of some spices and vegetables with food applications are used as/with other MPs in SSA for CVDs’ treatment; (4) non-animal protein is noted to be more effective than animal protein in reducing blood pressure [40]; (5) a diet rich in plant-based proteins has been recommended by nutritionists and relevant agencies [41] and (6) the potential of some AAs such as arginine, glutamine, glycine, leucine and taurine [5,27,42] to reduce atherogenic effects/CVDs risk has been indicated.

## 2. Materials and Methods

### 2.1. Collection and Preparation of Samples

Based on the literature, different plants’ parts of eight MPs used for CVDs and related risk factors’ treatment in SSA, some of which have found food applications, were investigated. These MPs’ parts were purchased at Odopetu market, Akure, Ondo State, Nigeria, with identification and authentication at the Department of Crop, Soil and Pest Technology, The Federal University of Technology, Akure, Ondo State, Nigeria. Details of the selected MPs and additional information regarding their key bioactive compounds are provided in Table 1 and Table 2, respectively.

After collection, the selected samples were washed, dried and ground to a powder with the use of a Binatone grinder (BLG 450) or a hammer mill (Changzhou, China) as appropriate. The powdered samples were then stored at −20 °C prior to FTIR and AAs analyses.

### 2.2. FTIR Spectroscopy Analysis

For the FTIR analysis, dried powdered samples of each of the selected MPs’ parts were loaded directly onto the FTIR spectroscope [112] and the spectra recorded in the middle infrared (MIR) region of 4000 cm^−1^ and 650 cm^−1^ [113]. PerkinElmer Spectrum 100 FTIR spectrometer was used for this purpose with a universal ATR sampling accessory. Scanning was performed at room temperature (25 ± 2 °C), while the background spectra collected under the same experimental conditions were subtracted from the sample spectra.

### 2.3. Determination of Crude Protein Content

The micro-Kjeldahl method [114] was used in determining the nitrogen content of the MPs’ parts powdered samples. With respect to Salo-väänänen and Koivistoinen [115] who noted the possibility of overestimating the true protein content of foods and other biological materials using a default conversion factor, the average nitrogen-to-protein conversion factor of 4.40 provided by Mariotti et al. [116] for vegetables, mushrooms and leaf proteins was used in converting the percentage nitrogen to crude protein.

### 2.4. Determination of Amino Acids Profile

The method as described by Adeyeye [117] with slight modifications was used in determining the AAs profile, except for tryptophan, of the selected MPs’ parts powdered samples. This involved defatting of the dried samples (about 2.0 g) using a Soxhlet apparatus with chloroform/ethanol mixture at a ratio of 2:1. Thereafter, hydrolysis of the defatted samples, involving seven milliliters of 6 M hydrochloric acid, was carried out, followed by evaporation of the filtrate to dryness in a rotary evaporator. Each residue of the MPs’ parts samples was then dissolved with 5 mL of acetate buffer (pH 2.0) and cool stored in a plastic specimen bottle at −20 °C.

For tryptophan determination, the method provided by Yust et al. [118] and Oriolowo et al. [119], involving alkaline hydrolysis with 4.2 M sodium hydroxide as well as neutralization of the hydrolysates to pH 7, was employed. Quantitative analysis of the amino acids in the hydolysates was achieved via the use of Applied Biosystems PTH Amino Acid Analyzer (Applied Biosystems Inc., Waltham, MA, USA) equipped with 2.1 mm ID × 220 mm cartridge columns packed with a reverse-phase support (PTH-C18). Norleucine was used as the internal standard.

### 2.5. Quantification and Estimation of Protein Quality

From the results obtained, the total AAs (TAAs), total EAAs (TEAAs), total NEAAs (TNEAAs), total acidic AAs (TAAAs) comprising glutamic and aspartic acids [117], total basic AAs (TBAAs) involving lysine, arginine and histidine [120], total neutral AAs (TNAAs) calculated as TAAs—(TAAAs + TBAAs), total sulfur AAs (TSAAs) from methionine and cysteine levels [18], percentage cysteine in TSAAs, total aromatic AAs (TArAAs) based on phenylalanine and tyrosine contents [18], as well as their percentages, were estimated. In addition, the percentages and ratio of TEAAs/TNEAAs [121,122], total BCAAs (TBCAAs), leucine to isoleucine (Leu/Ile) ratio [114], percentage of bitter AAs from the sum of leucine, valine, histidine, isoleucine, phenylalanine, methionine and tryptophan [123], savory (umami) amino AAs—glutamic and aspartic acids [122,123], sweet (threonine, serine, glycine and alanine) AAs [123] and the most limiting AAs were evaluated.

One of the equations of Alsmeyer and other researchers employed by Adeyeye et al. [114] and Kowalczewski et al. [124] (see Equation (1) below) was used in determining the predicted protein efficiency ratio (P-PER) of the MPs’ parts based on their AAs composition.

i.e., P-PER = −0.468 + 0.454 (Leu) − 0.105 (Tyr)(1)

In contrast, the slightly modified procedure of Tan et al. [123], involving the application of Equation (2), was followed for the amino acid scores’ (AAS) determination. For this, the FAO [125] recommended amino acid scoring patterns for young children (6 months to 3 years) as well as those for older children, adolescents and adults were also employed. The AAS were expressed as a ratio to unity, rather than in percentage, as recommended by the FAO/WHO [126].

(2) $$\mathrm{Amino}\;\mathrm{acid}\;\mathrm{score},\;\mathrm{AAS}=\frac{\mathrm{concentration}\;\mathrm{of}\;\mathrm{amino}\;\mathrm{acid}\;\left(\frac{\mathrm{mg}}{g}\right)\mathrm{in}\;\mathrm{the}\;\mathrm{medicinal}\;\mathrm{plants}{}^{’}\;\mathrm{parts}}{\mathrm{scoring}\;\mathrm{pattern}\;\left(\frac{\mathrm{mg}}{g}\right)\mathrm{protein}\;\mathrm{requirement}}$$

### 2.6. Statistical Analyses

The AAs, including EAAs, NEAAs, BCAAs and ArAAs, content of eight SSA MPs’ parts used for CVDs’ and related risk factors’ treatment were assessed with their AAS. One-way Analysis of Variance (SPSS^®^, version 26, IBM Statistics for Windows, New York, NY, USA) of the results obtained at 95% confidence level with Tukey’s *post-hoc* test was carried out. A readily available web tool for visualizing clustering of multivariate data, ClustVis (https://biit.cs.ut.ee/clustvis (accessed on 3 March 2021) [127]), was used for generating the heatmap, while principal component analysis was achieved by means of JMP^®^ Statistical Discovery^TM^ software, version 14 (SAS Institute Inc., Cary, NC, USA).

## 3. Results and Discussion

### 3.1. FTIR Analysis

**Figure 1:**
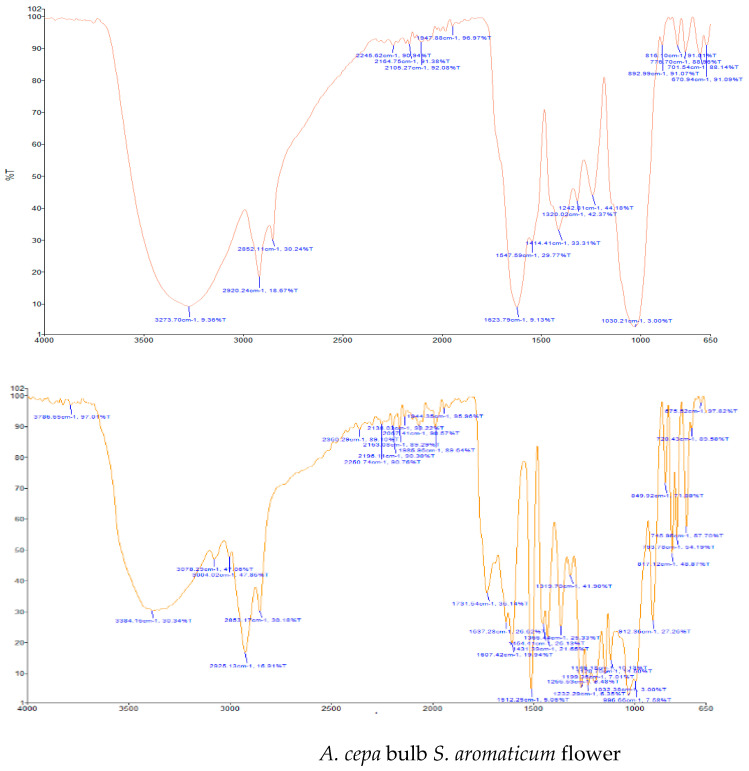

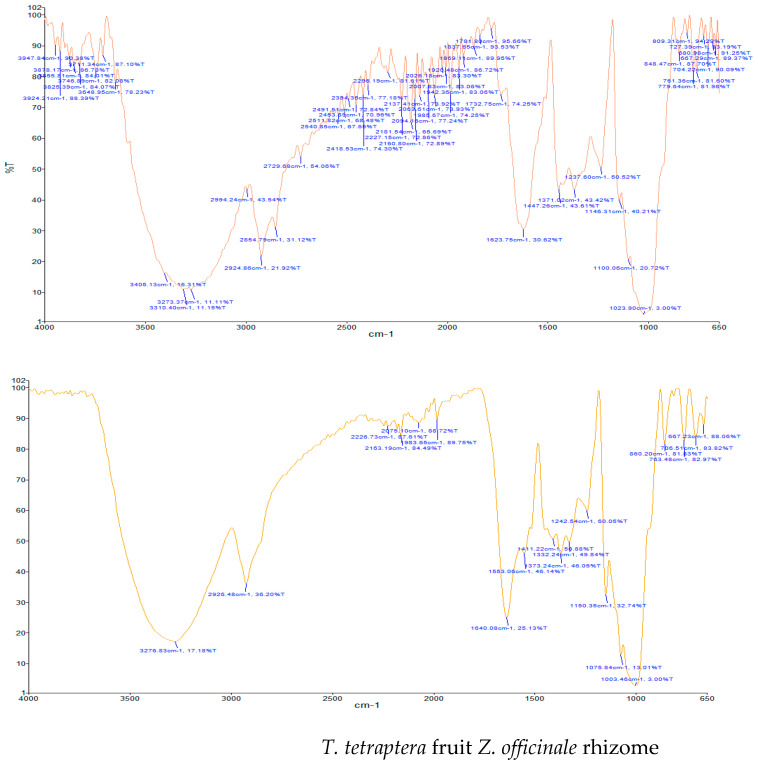
Fourier-transform infrared spectra of the studied parts with medicinal food plant applications.

**Figure 2:**
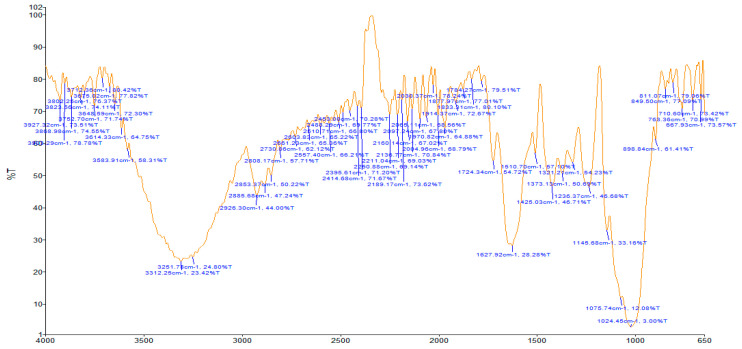

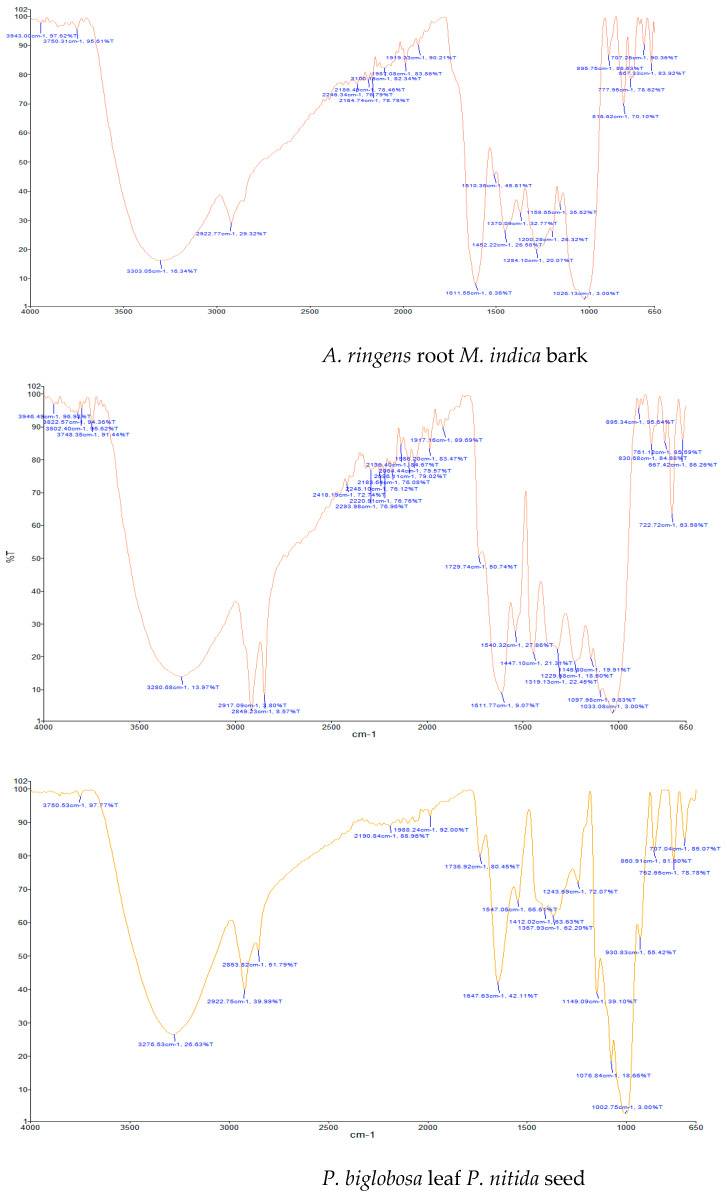
Fourier-transform infrared spectra of the studied parts used solely for medicinal applications.

Results of the FTIR analysis showing the different functional groups of the metabolites present in the assessed MPs’ parts are illustrated in Figure 1 and Figure 2, while details of the absorption bands and wave number of the dominant peaks in the functional group region are provided in Table 3. FTIR spectra of the samples established the presence of different bioactive functional groups such as -NH_2_, NH, as well as -OH, -NO_2_ and -CHO, among others, in the phytochemicals of these MPs’ parts. Similar to a study by Poojary et al. [128], they all showed the presence of a broad peak for hydrogen bonded -OH stretching in the diagnostic region, which is also seen in all the chemical structures in Table 2.

The presence of a hydroxyl (-OH) functional group is a fundamental part of most of the phenolic phytochemicals such as flavonoids and tannins [128] and would have contributed to the reported antioxidants as well as antidiabetic properties [35] of these MPs’ parts. Generally, the recorded functional groups, such as the nitro compound, alkyl group, alcohol, diene, aldehyde, vinyl group, carboxylic acid/derivative, alkene, primary and secondary amines in the assessed MPs’ parts, confirm the presence of secondary metabolites such as alkaloids, flavonoids, tannins and polyphenols [31,128], which explains their use in traditional medicine in different parts of SSA. For instance, Geetha et al. [31] linked the consumption of diets rich in polyphenols with protection against the development of diabetes, CVDs and some other diseases.

### 3.2. Crude Protein Content and Amino Acids Profile

**Figure 3:**
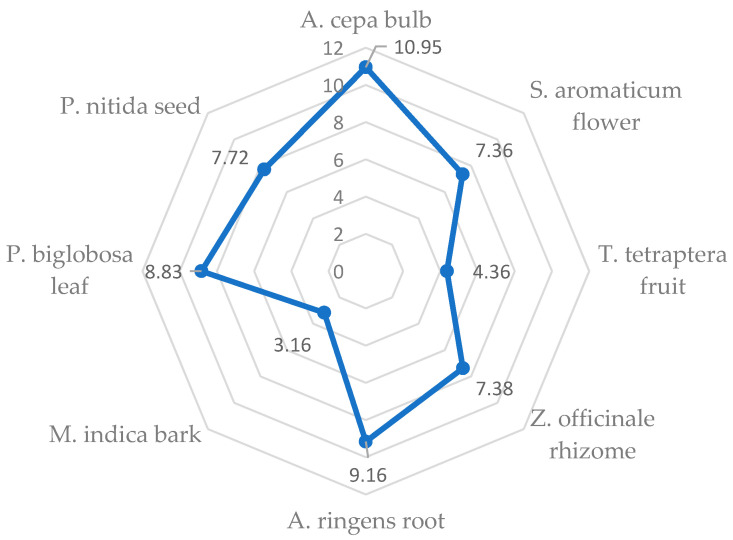
Levels of crude protein (g/100 g) in the selected medicinal plants’ parts.

Protein is required for growth and other functions of the body [122], while Odukoya [29] identified plants as the chief source of proteins consumed by humans. Outcome of the statistical analysis revealed that out of the eight investigated MPs’ plants, the bulb of *A. cepa*, which has been used as a food, spice and medicinal plant [82,129] for hyperlipidemia and CVD prevention [130], had the significantly highest (*p* < 0.05) crude protein content (10.95 g/100 g), while *M. indica* bark had the least (3.16 g/100 g) (Figure 3). In line with Odukoya et al. [131], this suggests that *A. cepa* bulb may have the highest concentration of EAAs among the studied MPs’ parts. Notwithstanding, the relatively low protein content of the *A. cepa* bulb when compared to other food sources agrees with Odukoya [29], who pointed out that vegetables have low protein contents.

#### Amino Acids

Results of the AA composition of the selected MPs’ parts that aid the assessment of their quality/value [132] are shown in Table 4 (for EAAs) and Table 5 (for NEAAs). For the EAAs, *A. cepa* was found to have the significantly (*p* < 0.05) highest concentration of histidine, lysine and phenylalanine (Table 4). The bulb of this MP, i.e., *A. cepa* and *P. biglobosa* leaf (solely used for medicinal purpose), also recorded the highest level of isoleucine and leucine. *S. aromaticum* flower, *M. indica* bark and *P. biglobosa* leaf had the highest methionine, threonine and valine contents, respectively, while the greatest amount of tryptophan was in *A. ringens* root and *P. nitida* seed.

Two MPs—*A. cepa* (aspartic acid and tyrosine) as well as *P. nitida* (alanine and proline)—singly had the highest concentration of two of the NEAAs. In contrast, the greatest amount of arginine (*A. cepa* and *P. biglobosa*), cysteine (*M. indica* and *P. biglobosa*) and glutamic acid (*A. cepa* and *S. aromaticum*) was found in two of the studied MPs. The significantly highest level of serine (*p* < 0.05) was recorded in *A. cepa*, *S. aromaticum* and *P. nitida*, while *Z. officinale* and *M. indica* had the lowest glycine contents. A relatively high value of arginine, which is reported to be vital for children [117], was noted in *A. cepa*.

The results of the analyses are in agreement with a study by Poggiogalle et al. [133], where vegetable proteins were reported to be rich sources of glutamic acid. They are also in line with Olsen et al. [23], who pointed out that plants are poor sources of the sulfur AAs, methionine and cysteine. With respect to Ntuli [134], the significantly highest concentration of hydrophilic AAs (histidine, lysine and tyrosine) and the relatively high concentration of cysteine in *A. cepa*, in a way, explains the semi-succulence and soft-textured nature of the bulb [134]. As reported by this author, i.e., Ntuli [134], the presence of cysteine and tyrosine (an ArAA) in *A. cepa* will contribute to its antioxidant activities when found in certain peptides chains.

**Figure 4:**
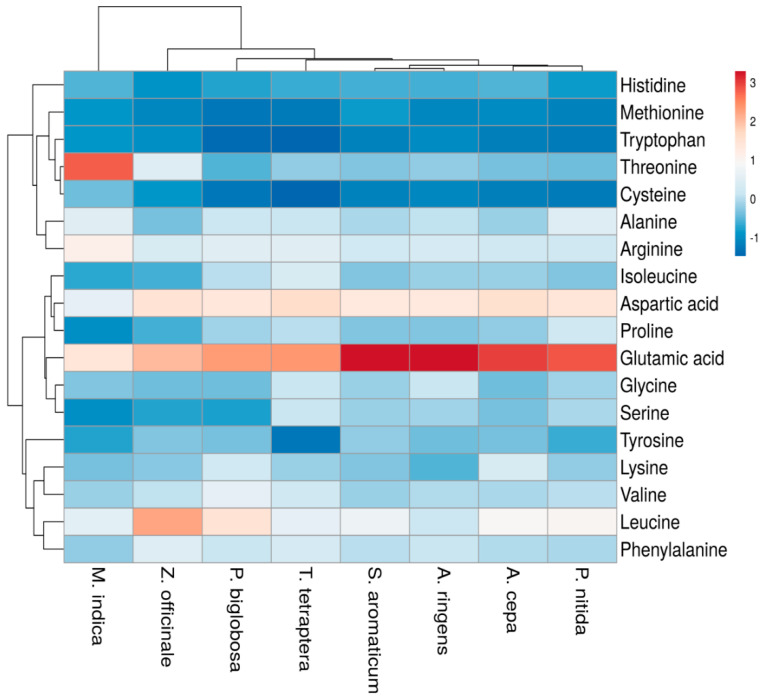
Heatmap reflecting the concentration of amino acids (essential and non-essential) in the selected medicinal plants’ parts. Color of the heatmap ranging from deep red to deep blue (i.e., scale 3 to −1) indicates higher to lower concentration of the amino acids.

Generally, as seen in the heatmap (Figure 4), glutamic acid and aspartic acid were the most abundant AAs in most of the tested MPs’ parts. Although they are both NEAAs (Table 5), glutamic acid is vital for optimal organ functioning [135]. The research outcome is consistent with a study by Fredotovic et al. [136], where glutamic acid was the second most abundant AA in *A. cepa*, and that by Neves et al. [137], where it was reported that these two NEAAs (glutamic acid and aspartic acid) were the most common AAs in the examined jambu (*Acmella oleracea*) and several vegetables. They were also the most abundant AAs in the two accessions of *Amaranthus cruentus* seeds flour examined by Esan et al. [138] as well as fish samples studied by Adeyeye et al. [117]. The two NEAAs impart acidic characteristics to proteins [134], act as a neurotransmitter as well as contribute to energy production, transamination, insulin regulation and the formation of other AAs [137]. Lopez et al. [139] indicated their importance in the food industry based on their respective role in hormonal regulation and immunological stimulation. Glutamic acid is also reported to be the key “umami” substrate underlying the unique taste of *A. cepa* [136].

As clearly observed in the heatmap (Figure 4), leucine was the most abundant AA in *Z. officinale* rhizome, which contributes to the imbalance of its Leu/Ile ratio discussed in Section 3.3.

### 3.3. Protein Quality

According to Elhardallou et al. [140] and Sun et al. [141], the quality of proteins depends on their AA composition and proportion. Among the tested MPs’ parts, *A. cepa* bulb had the significantly highest level (*p* < 0.05) of TAAs, TNEAAs, TNAAs, TAAAs and TBAAs; the greatest amounts of TEAAs (with or without histidine) and P-PER were also recorded in this MP (*A. cepa* bulb) as well as *P. biglobosa* leaf (Table 6). This latter MP, i.e., *P. biglobosa* leaf, had the highest concentration of TBCAAs. The highest percentages of TNAAs (*T. tetraptera*, *Z. officinale* and *M. indica*), TAAAs (*S. aromaticum*) and TBAAs (*A. cepa*, *M. indica* and *P. biglobosa*) with respect to the TAAs were also observed in some of these MPs’ parts. Surprisingly, *M. indica* bark had the highest TEAAs/TNEAAs ratio, percentage of cysteine in TSAAs and percentage of sweet AAs.

The richest TSAAs was found in *S. aromaticum* flower and *P. bigblobosa* leaf, while the highest level of TArAAs was noted in these MPs (i.e., *S. aromaticum* and *P. bigblobosa*) and *A. cepa* bulb. Meanwhile, *Z. officinale* rhizome had the highest Leu/Ile ratio of 4.09. This medicinal plant (*Z. officinale*) and *P. biglobosa* also had the greatest percentage of bitter AAs, whereas *S. aromaticum* recorded the highest percentage of savory (umami) AAs.

The outcome of the experiment revealed that although *A. cepa* bulb had the richest level of AAs, all the studied MPs’ parts had a TEAAs/TAAs percentage content higher than the 36% considered appropriate for an ideal protein [134,138,142]. Notwithstanding, as reported by Parniakov et al. [143], their TEAAs/TNEAAs ratio being less than unity (< 1) showed that they are not good sources of EAAs. *P. biglobosa* leaf, followed by *A. cepa* bulb, had the highest concentration of total BCAAs (leucine, isoleucine and valine), which, according to Jin et al. [144], have antioxidant potentials and play other important roles in the body. In addition, the results of the investigation agree with Tobias et al. [145] that vegetable proteins are also sources of BCAAs. With respect to Esan et al. [138], the percentage of total acidic AAs (TAAAs (%)) of all the tested MPs’ parts greater than that of total basic AAs (TBAAs (%)) indicates that the protein of these plants is chiefly acidic in nature.

Meanwhile, Mendoza [146] noted that cysteine can positively affect mineral absorption and, according to Adeyeye et al. [114,117], most animal proteins have a lower level of this sulfur AA (cysteine) than methionine, in which the reverse is the case in many vegetable proteins. Thus, with respect to the latter authors, *M. indica* bark, with the significantly (*p* < 0.05) highest percentage of cysteine in TSAA (69.04%), can be likened to plant proteins, whereas five of the studied MPs’ (*A. cepa*, *S. aromaticum*, *T. tetraptera*, *P. biglobosa* and *P. nitida*) parts with a percentage of cysteine in TSAAs less than 50% is similar to those of animal proteins, as Adeyeye et al. [114] reported that cysteine is unlikely to contribute more than 50% of the total SAAs in animal protein. Adeyeye et al. [114] also pointed out that cysteine and tyrosine can supply up to 33.33% of the need for TSAAs and TArAAs, respectively.

According to Kowalczewski et al. [124], PER can be used to assess the nutritional value of a protein in which a value greater than two indicates the high quality of the protein. In this study, *A. cepa* bulb and *P. biglobosa* leaf were found to have the significantly highest P-PER. In line with Kowalczewski et al. [124], the P-PER values of these two MPs’ parts (i.e., *A. cepa* bulb and *P. biglobosa* leaf) being > 2 suggest that only their protein is of high quality. In contrast, the significantly highest Leu/Ile ratio in *Z. officinale* rhizome of 4.09 suggests that the excessive intake of this MP, when included as part of a human diet, may contribute to pellagra, as an amino acid imbalance from excess leucine has been connected to the development of this disease because high leucine in the diet impairs tryptophan and niacin metabolism [114]. The results of the major taste components (bitter AAs (%), savory AAs (%) and sweet AAs (%)) of the assessed MPs’ parts agree with Lisiewska et al. [147] that AAs influence the sensory attribute of products.

#### Amino Acid Score (AAS)

Following the FAO recommended [125] amino acid scoring patterns for young children (6 months to 3 years) as well as for older children, adolescents and adults, a similar statistical result obtained for the EAAs was recorded for the AAS of histidine, the BCAAs (isoleucine, leucine and valine), lysine, threonine and valine in the selected MPs’ parts, as seen in Table 7 and Table 8. The AAS of methionine and cysteine, sulfur AAs [18,23,134,148], based on the two scoring patterns, was highest in *S. aromaticum* and *P. biglobosa*, while these two MPs with *A. cepa* also had the greatest AAS for phenylalanine and tyrosine, aromatic AAs [18].

According to the FAO/WHO [126], the limiting AA (LAA) is the EAA present in the lowest proportion when compared to the same quantity of the standard protein, while Neves et al. [137] noted that scores less than unity pinpoint the LAAs. As shown in Table 7 and Table 8, lysine was the most limiting AA in almost all the assessed MPs’ parts. This is in line with the research finding of Lisiewska et al. [147], where, in relation to protein quality, lysine was the first LAA in the studied kale leaves. The present study’s experimental results also agree with the FAO/WHO [126], which noted that lysine is usually the first-limiting AA in many food sources. Adeyeye et al. [117] also reported that the EAAs that often act in a limiting capacity are lysine, methionine and cysteine, threonine as well as tryptophan. This explains the selection of lysine in Table 8 as the real LAA in *M. indica*. Among all the studied MPs’ parts, only *A. cepa* bulb had no LAA for older children, adolescents and adults. Surprisingly, *Z. officinale* rhizome, with food application, was found to be limiting in all the AAs considered for all the age categories.

To a great extent, the AAS results in Table 7 and Table 8 agree with Bleakley and Hayes [41] as well as Marti-Quijal et al. [121] that plant proteins are often an incomplete protein source as they usually lack one or more EAAs. Hence, aside from *A. cepa* bulb, all the other studied MPs with food applications (though not consumed majorly as food) are to be combined with other protein sources to achieve the AA requirements in human nutrition [137].

### 3.4. Principal Component Analysis (PCA)

**Figure 5:**
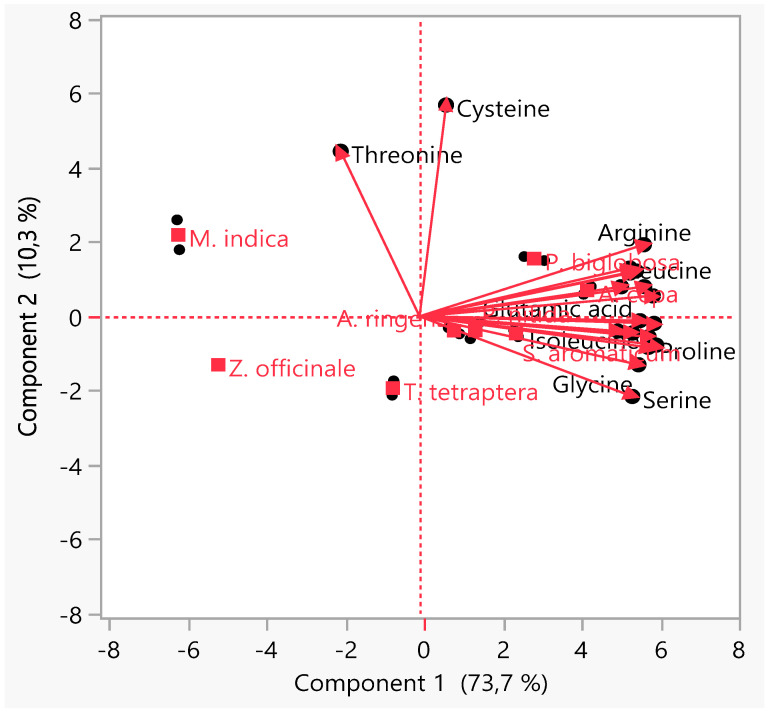
Principal component analysis of the amino acids composition of the selected medicinal plants’ parts.

PCA, an unsupervised clustering/display method, was used to check the similarities, hidden patterns and outliers in the data set obtained as well as reduce the dimensionality [51,149,150]. The biplot from the PCA (Figure 5) showed two principal components, PC1 and PC2, describing 84% of the variation. As seen in quadrant two, there is a close association between the AAs’ composition of *A. cepa* bulb and *P. biglobosa* leaf with two AAs (leucine and arginine) contributing principally to the separation of the clusters of these two MPs from those of the others. Meanwhile, the clusters of *M. indica* bark in quadrant one and *Z. officinale* rhizome in quadrant three reflect the difference in the AA content of these MPs when compared with the observed levels in the other studied MPs. To a large extent, the PCA revealed that there is no marked difference between the AA composition of MPs that have found food and medicinal applications from those solely used for medicinal purposes.

## 4. Conclusions

The current study provides the required information on the AA distribution as well as the phytochemicals profile of different MPs’ parts used in SSA for the treatment of CVDs and their associated risk factors. FTIR analysis confirmed the presence of hydroxyl, nitro, amino and other functional groups in the bioactive compounds of the assessed MPs’ parts. Despite the highest crude protein and TAA contents recorded in *A. cepa* bulb, including the noted quality of its protein, analysis of the TEAAs/TNEAAs ratio revealed that all the studied MPs’ parts are not good sources of EAAs. An investigation on their percentage TAAAs and TBAAs showed that their protein is chiefly acidic in nature, while *Z. officinale* rhizome, with food application, was found to be limiting in all the AAs considered for all the age categories.

Generally, the study indicated that the use of any of the selected MPs’ parts for CVDs and related risk factors’ treatment in SSA, vis-à-vis their AA composition, is less likely to contribute to an elevation in the circulation of BCAAs and other AAs in a way that would affect human health negatively. It also affirmed that although plants may be incomplete protein sources lacking one or more EAAs, some are rich sources of medicinally important phytochemicals that can assist in the discovery of new drugs for CVDs’ treatment. The inclusion of *A. cepa* bulb in food for patients (adults) suffering from CVDs and their risk factors is encouraged, while excessive intake of *Z. officinale* rhizome (Leu/Ile ratio = 4.09) in the human diet or as a traditional home remedy should be avoided to prevent pellagra development.

## Figures and Tables

**Table 1:** Details of the selected sub-Saharan African medicinal plants.

S/N	Medicinal Plants	Family	English Name	Plant’s Part Used in the Current Study	Ailments Used for	Countries in SSA Where Use Has Been Reported	References
***Studied part with medicinal food plant applications***							
1	*Allium cepa*	Amaryllidaceae	Onion	Bulb	Cardiovascular disease, Diabetes, High cholesterol level, Hypertension	Benin, Burkina Faso Cameroon, DR Congo Eritrea, Ethiopia Gabon, Mauritius, Nigeria, Sudan, Togo	[43,44,45,46,47,48,49,50,51,52,53,54,55,56,57,58,59]
2	*Syzgium aromaticum* L.	Myrtaceae	Clove bud/Clove	Flower	Diabetes, Hypertension	Nigeria	[47,51,60]
3	*Tetrapleura tetraptera*	Fabaceae	Ring worm bush	Fruit	Cardiovascular activities, Diabetes, Hypertension	Benin, Cameroon Gabon, Ghana, Nigeria	[43,51,53,58,60,61,62]
4	*Zingiber officinale*	Zingiberaceae	Ginger	Rhizome	Diabetes, High cholesterol level, Hypertension	Benin, Eritrea, Gabon, Mauritius, Nigeria	[43,47,51,57,58,59,63,64]
***Studied part used solely for medicinal applications***							
5	*Aristolochia ringens* Vahl.	Aristochiaceae	Pelican flower	Root	Diabetes, Heart attack	Nigeria	[48,51,60]
6	*Mangifera indica* L.	Anacardiaceae	Mango	Bark	Diabetes, Hypertension	Benin, Cameroon, DR Congo, Eritrea, Gabon, Ghana, Guinea, Kenya, Mauritius, Nigeria, Togo, Zambia, Zimbabwe	[43,47,51,54,55,56,57,58,59,60,65,66,67,68,69,70,71,72]
7	*Parkia biglobosa* Benth.	Fabaceae	African locust bean	Leaf	Diabetes, Hypertension, Heart disorders	Benin, Burkina Faso, Cote d’Ivoire, Ghana, Nigeria, Togo	[47,50,51,52,61,66,70,73,74,75,76]
8	*Picralima nitida*	Apocynaceae	Picralima	Seed	Cardiovascular diseases, Diabetes, Hypertension	Benin, Gabon, Ghana Nigeria, Togo	[43,50,51,58,60,61,70,77,78]

**Table 2:** Key bioactive compounds in the selected sub-Saharan African medicinal plants based on the literature.

S/N	Medicinal Plants	Key Bioactive Compounds	Chemical Structure	Influence on Cardiovascular Diseases and Their Risk Factors	References
1	*Allium cepa*	Flavonoids (particularly flavonols), frutooligosaccharides and sulfur compounds. Characterized for its flavonol quercetin and quercetin derivates.	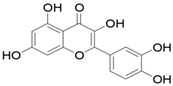 Quercetin	Inhibit platelet aggregation. Reduce serum triglycerides and cholesterol levels. Alleviate hyperglycemia.	[79,80,81,82,83]
2	*Syzgium aromaticum* L.	Eugenol	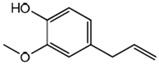 Eugenol: 4-allyl-2-methoxyphenol	Inhibits platelet aggregation. Reduce serum triglycerides and cholesterol levels.	[84,85,86,87,88]
3	*Tetrapleura tetraptera*	Saponin triterpenes, a triterpene glycoside (aridanin) and a coumarin (scopoletin).	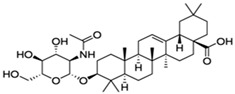 Aridanin (an *N*-acetylglycoside of oleanolic acid)	Lowers blood glucose level.	[89,90,91,92,93,94]
4	*Zingiber officinale*	*Terpenes*: β-Bisabolene, α-Curcumene, Zingiberene, α-Farnesene and β-Sesquiphellandrene. *Phenolic compounds*: Gingerols (e.g., 6-gingerol), Shogaols (e.g., 6-shogaol) and Paradols (e.g., 6-paradol)	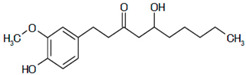 6-Gingerol: Often used as an indicator of ginger quality	Inhibits platelet aggregation. Reduce the levels of blood lipids and blood pressure.	[95,96,97,98]
5	*Aristolochia ringens* Vahl.	Dianoside G, Trilobine, Asiatic acid, Magnoflorine, Quercetin 3-*O*-glucuronide and Strictosidine.	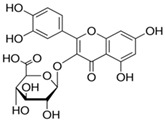 Quercetin 3-*O*-glucuronide (A flavonol glucuronide)	Reduce blood glucose level.	[99,100]
6	*Mangifera indica* L.	Gallotanins, Gallic acid and its derivatives, Mangiferin, Flavonoids, Catechin and Phenolic acids	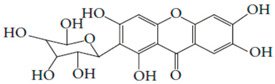 Mangiferin (2-β-D-glucopyranosyl-1, 3, 6, 7-tetrahydroxyxanthone): Major component in mango stem bark extract	Reduce serum total cholesterol level and glucose absorption.	[101,102,103,104,105]
7	*Parkia biglobosa* Benth.	Flavonoids (catechin, epigallocatechin, epigallocatechin gallate, quercetin, rutin and kaempferol) and Phenolic acids (gallic, chlorogenic and caffeic acids)	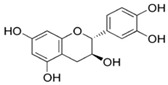 Catechin: A flavanol	Serum cholesterol lowering activity	[106,107,108,109]
8	*Picralima nitida*	Indole Alkaloids: akuammine, akuammidine, akuammicine, akuammigine and pseudoakuammigine	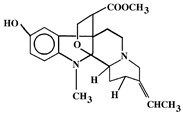 Akuammine: Most abundant alkaloid in the seed	Reduce glycaemia	[110,111]

**Table 3:** Major absorption bands observed in the functional group region of the selected medicinal plants’ parts’ FTIR spectra.

Medicinal Plants	Absorption Peak (cm^−1^) (Vibration Type)	Functional Group Assignment
***Studied part with medicinal food plant applications***		
*A. cepa*	1547.59 (N-O bend); 1623.79 (N-H bend); 2852.11 (C-H stretch); 2920.24 (C-H stretch); 3273.70 (O-H stretch)	Nitro compound; Primary amine; Alkyl group; Alkyl group; Alcohol
*S. aromaticum*	1512.25 (N-O bend); 1607.42 (C=C bend); 1637.28 (N-H bend); 1731.54 (C=O bend); 2853.17 (C-H stretch); 2925.13 (C-H stretch); 3004.02 (C-H stretch); 3078.29 (N-H stretch); 3384.16 (O-H stretch)	Nitro compound; Diene; Primary amine; Aldehyde; Alkyl group; Alkyl group; Alkyl group; Secondary amine; Alcohol
*T. tetraptera*	1623.75 (C=C bend); 2854.79 (C-H stretch); 2924.86 (C-H stretch); 299.24 (C-H stretch); 3273.37 (O-H stretch); 3310.40 (O-H stretch); 3405.13 (O-H stretch)	Diene; Alkyl group; Alkyl group; Vinyl group; Alcohol; Alcohol; Alcohol
*Z. officinale*	1553.06 (C=O bend); 1640.08 (N-H bend); 2926.48 (C-H stretch); 3276.83 (O-H stretch)	Carboxylic acid/derivative; Primary amine; Alkyl group; Alcohol
***Studied part used solely for medicinal applications***		
*A. ringens*	1627.92 (N-H bend); 2853.37 (C-H stretch); 2885.68 (C-H stretch); 2926.30 (C-H stretch); 3251.78 (O-H stretch); 3312.25 (O-H stretch)	Primary amine; Alkyl group; Alkyl group; Alkyl group; Alcohol; Alcohol
*M. indica*	1510.35 (N-O bend); 1611.55 (N-H bend); 2922.77 (C-H stretch); 3303.05 (O-H stretch)	Nitro compound; Primary amine; Alkyl group; Alcohol
*P. biglobosa*	1540.32 (N-O bend); 1611.77 (N-H bend); 1729.74 (C=O bend); 2849.23 (C-H stretch); 2917.09 (C-H stretch); 3280.68 (O-H stretch)	Nitro compound; Primary amine; Aldehyde; Alkyl group; Alkyl group; Alcohol
*P. nitida*	1647.63 (C=C bend); 2853.82 (C-H stretch); 2922.75 (C-H stretch); 3276.53 (O-H stretch)	Alkene; Alkyl group; Alkyl group; Alcohol

**Table 4:** Essential amino acids profile (mg/g crude protein) of the selected medicinal plants’ parts.

Medicinal Plants	His	Ile	Leu	Lys	Met	Phe	Thr	Try	Val
***Studied Part with Medicinal Food Plant Applications***									
*A. cepa*	28.10 ^a^ ± 0.00	40.80 ^a^ ± 0.50	74.40 ^a^ ± 0.30	56.60 ^a^ ± 0.10	13.70 ^b^ ± 0.20	44.30 ^a^ ± 0.00	34.70 ^a,b^ ± 2.35	9.70 ^a,b^ ± 0.30	43.50 ^b^ ± 0.20
*S. aromaticum*	22.35 ^b,c^ ± 0.65	31.60 ^c^ ± 0.50	60.45 ^b^ ± 1.45	31.00 ^c^ ± 0.30	16.65 ^a^ ± 0.45	40.35 ^b^ ± 0.45	30.80 ^a,b^ ± 0.80	8.00 ^b,c^ ± 0.10	35.50 ^c,d^ ± 0.50
*T. tetraptera*	18.70 ^d^ ± 0.50	34.35 ^b^ ± 0.35	37.60 ^e^ ± 0.90	26.10 ^c^ ± 0.10	10.70 ^c^ ± 0.50	33.25 ^d^ ± 0.45	25.20 ^b^ ± 0.80	6.95 ^c,d^ ± 0.15	31.70 ^e^ ± 0.20
*Z. officinale*	6.25 ^f^ ± 0.15	10.65 ^e^ ± 0.15	43.50 ^d^ ± 0.30	14.55 ^d,e^ ± 0.25	4.95 ^e^ ± 0.15	22.60 ^e^ ± 0.40	23.05 ^b^ ± 0.25	5.90 ^d,e^ ± 0.10	18.10 ^f^ ± 0.00
***Studied Part Used Solely for Medicinal Applications***									
*A. ringens*	20.75 ^c^ ± 0.35	30.60 ^c^ ± 0.50	37.95 ^e^ ± 0.55	21.48 ^c,d^ ± 2.42	8.55 ^d^ ± 0.55	37.65 ^c^ ± 0.45	28.85 ^b^ ± 0.55	10.25 ^a^ ± 0.25	33.75 ^d^ ± 0.15
*M. indica*	9.05 ^e^ ± 0.15	7.05 ^f^ ± 0.15	23.35 ^f^ ± 0.55	11.35 ^e^ ± 0.25	5.05 ^e^ ± 0.25	13.75 ^f^ ± 0.45	52.60 ^a^ ± 2.33	5.00 ^e^ ± 0.80	14.30 ^g^ ± 0.30
*P. biglobosa*	23.30 ^b^ ± 0.30	39.75 ^a^ ± 0.15	72.10 ^a^ ± 0.30	45.75 ^b^ ± 0.65	12.15 ^b,c^ ± 0.15	42.15 ^a,b^ ± 0.45	27.75 ^b^ ± 2.23	8.50 ^a,b,c^ ± 0.10	52.60 ^a^ ± 0.60
*P. nitida*	18.20 ^d^ ± 0.30	28.50 ^d^ ± 0.30	55.40 ^c^ ± 0.60	31.00 ^c^ ± 0.80	11.50 ^c^ ± 0.30	34.15 ^d^ ± 0.45	26.95 ^b^ ± 0.85	10.25 ^a^ ± 0.25	36.00 ^c^ ± 0.00

Values represent mean ± standard error. Means followed by different letters are significantly different (*p <* 0.05) according to Tukey’s *post-hoc* test. His = histidine; Ile = isoleucine; Leu = leucine; Lys = lysine; Met = methionine; Phe = phenylalanine; Thr = threonine; Try = tryptophan; Val = valine.

**Table 5:** Non-essential amino acids profile (mg/g crude protein) of the selected medicinal plants’ parts.

Medicinal Plants	Ala	Asp	Arg	Cys	Glu	Gly	Pro	Ser	Tyr
***Studied Part with Medicinal Food Plant Applications***									
*A. cepa*	40.20 ^b,c^ ± 0.00	92.79 ^a^ ± 1.68	51.60 ^a^ ± 0.00	9.40 ^b^ ± 0.30	136.25 ^a^ ± 2.52	33.50 ^a^ ± 1.70	38.60 ^a,b^ ± 0.00	35.15 ^a^ ± 2.15	34.40 ^a^ ± 0.00
*S. aromaticum*	37.15 ^c,d^ ± 0.75	77.70 ^a,b^ ± 0.80	46.85 ^b^ ± 0.45	8.20 ^b,c^ ± 0.30	132.85 ^a^ ± 0.35	35.15 ^a^ ± 0.95	30.50 ^c^ ± 0.00	35.65 ^a^ ± 0.55	33.55 ^a,b^ ± 0.85
*T. tetraptera*	30.70 ^e^ ± 0.40	53.05 ^c^ ± 0.95	36.55 ^d^ ± 0.45	6.40 ^d^ ± 0.30	64.75 ^c^ ± 1.15	30.90 ^a^ ± 0.70	28.90 ^c,d^ ± 0.50	30.65 ^a,b^ ± 0.45	9.45 ^e^ ± 0.85
*Z. officinale*	13.45 ^g^ ± 0.15	34.40 ^d^ ± 0.30	21.50 ^f^ ± 0.90	7.30 ^c,d^ ± 0.00	41.65 ^c,d^ ± 0.75	12.55 ^b^ ± 0.25	10.70 ^e^ ± 0.50	8.90 ^c^ ± 0.30	13.75 ^d,e^ ± 0.05
***Studied Part Used Solely for Medicinal Applications***									
*A. ringens*	36.20 ^d^ ± 0.20	65.40 ^b,c^ ± 0.30	42.15 ^c^ ± 0.85	9.40 ^b^ ± 0.30	112.00 ^a,b^ ± 0.00	36.55 ^a^ ± 0.45	27.40 ^d^ ± 1.00	30.95 ^a,b^ ± 0.15	24.95 ^b,c^ ± 0.85
*M. indica*	22.80 ^f^ ± 0.00	24.35 ^d^ ± 3.55	30.95 ^e^ ± 0.85	11.25 ^a^ ± 0.25	34.45 ^d^ ± 0.35	12.15 ^b^ ± 0.25	3.58 ^f^ ± 0.53	3.65 ^c^ ± 0.45	6.90 ^e^ ± 0.00
*P. biglobosa*	43.20 ^a,b^ ± 0.00	69.39 ^b,c^ ± 2.15	50.45 ^a^ ± 0.45	11.80 ^a^ ± 0.30	90.73 ^b^ ± 3.38	30.93 ^a^ ± 1.82	36.55 ^b^ ± 0.05	22.80 ^b^ ± 2.10	32.05 ^a,b^ ± 1.05
*P. nitida*	44.75 ^a^ ± 1.55	64.50 ^b,c^ ± 0.60	39.60 ^c,d^ ± 0.00	9.40 ^b^ ± 0.30	95.00 ^b^ ± 1.10	32.55 ^a^ ± 0.45	40.10 ^a^ ± 0.50	34.00 ^a^ ± 0.00	21.40 ^c,d^ ± 2.05

Values represent mean ± standard error. Means followed by different letters are significantly different (*p <* 0.05) according to Tukey’s *post-hoc* test. Ala = alanine; Asp = aspartic acid; Arg = arginine; Cys = cysteine; Glu = glutmamic acid; Gly = glycine; Pro = proline; Ser = serine; Tyr = tyrosine.

**Table 6:** Concentration (mg/g crude protein), percentage and ratio of specific groups of amino acids in the selected medicinal plants’ parts.

Groups of Amino Acids	Studied Part with Medicinal Food Plant Applications				Studied Part Used Solely for Medicinal Applications			
	*A. cepa*	*S. aromaticum*	*T. tetraptera*	*Z. officinale*	*A. ringens*	*M. indica*	*P. biglobosa*	*P. nitida*
Total amino acids (TAAs)	817.69 ^a^ ± 6.01	714.30 ^b^ ± 0.20	515.90 ^d^ ± 2.10	313.75 ^e^ ± 1.15	614.83 ^c^ ± 6.78	291.58 ^e^ ± 7.23	711.94 ^b^ ± 9.33	633.25 ^c^ ± 6.95
Total essential amino acids (TEAAs)								
*-With Histidine*	345.80 ^a^ ± 5.30	276.70 ^b^ ± 0.70	224.55 ^d^ ± 1.85	149.55 ^e^ ± 0.35	229.83 ^c,d^ ± 4.28	141.50 ^e^ ± 9.10	324.05 ^a^ ± 5.45	251.95 ^b,c^ ± 1.55
*-No Histidine*	317.70 ^a^ ± 5.30	254.35 ^b^ ± 0.05	205.85 ^d^ ± 1.35	143.30 ^e^ ± 0.20	209.08 ^c,d^ ± 4.63	132.45 ^e^ ± 8.95	300.75 ^a^ ± 5.75	233.75 ^b,c^ ± 1.85
TEAAs (%)								
*-With Histidine*	42.29 ^c,d,e^ ± 0.28	38.74 ^e,f^ ± 0.09	43.53 ^b,c,d^ ± 0.18	47.67 ^a,b^ ± 0.06	37.38 ^f^ ± 0.28	48.48 ^a^ ± 1.92	45.56 ^a,b,c^ ± 1.03	39.79 ^d,e,f^ ± 0.19
*-No Histidine*	38.86 ^b,c^ ± 0.21	35.61 ^c,d^ ± 0.00	39.90 ^b,c^ ± 0.09	45.67 ^a^ ± 0.10	34.00 ^d^ ± 0.38	45.38 ^a^ ± 1.95	42.28 ^a,b^ ± 0.85	36.91 ^c,d^ ± 0.11
Total non-essential amino acids (TNEAAs)	471.89 ^a^ ± 4.25	437.60 ^a,b^ ± 0.50	291.35 ^d^ ± 0.25	164.20 ^e^ ± 0.80	385.00 ^b,c^ ± 2.50	150.08 ^e^ ± 1.88	387.89 ^b,c^ ± 7.51	381.30 ^c^ ± 5.40
TNEAAs (%)	57.70 ^b,c,d^ ± 0.28	61.26 ^a,b^ ± 0.09	56.47 ^c,d,e^ ± 0.18	52.33 ^e,f^ ± 0.06	62.62 ^a^ ± 0.28	51.52 ^f^ ± 1.92	54.44 ^d,e,f^ ± 1.03	60.21 ^a,b,c^ ± 0.19
Ratio of TEAAs to TNEAAs								
*-With Histidine*	0.73 ^c,d,e^ ± 0.01	0.63 ^d,e^ ± 0.00	0.77 ^b,c,d^ ± 0.01	0.91 ^a,b^ ± 0.00	0.59 ^e^ ± 0.01	0.94 ^a^ ± 0.07	0.84 ^a,b,c^ ± 0.03	0.66 ^d,e^ ± 0.01
*-No Histidine*	0.67 ^b,c,d^ ± 0.01	0.58 ^c,d^ ± 0.00	0.71 ^b,c^ ± 0.00	0.87 ^a^ ± 0.00	0.54 ^d^ ± 0.01	0.88 ^a^ ± 0.07	0.78 ^a,b^ ± 0.03	0.61 ^c,d^ ± 0.00
Total branched-chain amino acids (TBCAAs)	158.70 ^b^ ± 0.60	127.55 ^c^ ± 1.45	103.65 ^e^ ± 1.45	72.25 ^f^ ± 0.15	102.30 ^e^ ± 0.20	44.70 ^g^ ± 0.70	164.45 ^a^ ± 0.15	119.90 ^d^ ± 0.30
TBCAAs (%)	19.42 ^b^ ± 0.35	17.86 ^b,c^ ± 0.21	20.09 ^b^ ± 0.19	23.03 ^a^ ± 0.04	16.64 ^c,d^ ± 0.15	15.35 ^d^ ± 0.62	23.13 ^a^ ± 0.89	18.94 ^b,c^ ± 0.26
Total neutral amino acids (TNAAs)	452.35 ^a^ ± 5.55	403.55 ^b,c^ ± 0.55	316.75 ^e^ ± 1.45	195.40 ^f^ ± 0.60	353.05 ^d^ ± 1.75	181.43 ^f^ ± 9.18	432.33 ^a,b^ ± 3.84	384.95 ^c,d^ ± 6.95
TNAAs (%)	55.33 ^c^ ± 0.54	56.49 ^c^ ± 0.06	61.39 ^a^ ± 0.03	62.28 ^a^ ± 0.04	57.43 ^b,c^ ± 0.35	62.18 ^a^ ± 1.61	60.76 ^a,b^ ± 0.77	60.79 ^a,b^ ± 0.43
Total acidic amino acids (TAAAs)	229.04 ^a^ ± 4.19	210.55 ^a,b^ ± 1.15	117.80 ^d,e^ ± 0.20	76.05 ^e,f^ ± 1.05	177.40 ^b,c^ ± 0.30	58.80 ^f^ ± 3.20	160.12 ^c^ ± 5.52	159.50 ^c,d^ ± 0.50
TAAAs (%)	27.99 ^a,b^ ± 0.92	29.48 ^a^ ± 0.17	22.83 ^c,d^ ± 0.13	24.24 ^b,c,d^ ± 0.25	28.86 ^a,b^ ± 0.37	20.21 ^d^ ± 1.59	22.43 ^c,d^ ± 1.44	25.19 ^a,b,c^ ± 0.36
Total basic amino acids (TBAAs)	136.30 ^a^ ± 0.10	100.20 ^c^ ± 0.80	81.35 ^d^ ± 0.85	42.30 ^e^ ± 0.50	84.38 ^d^ ± 5.33	51.35 ^e^ ± 1.25	119.50 ^b^ ± 0.10	88.80 ^d^ ± 0.50
TBAAs (%)	16.68 ^a^ ± 0.38	14.03 ^b,c^ ± 0.11	15.77 ^a,b^ ± 0.10	13.48 ^c^ ± 0.21	13.72 ^b,c^ ± 0.71	17.61 ^a^ ± 0.01	16.81 ^a^ ± 0.67	14.02 ^b,c^ ± 0.07
Total sulphur amino acids (TSAAs)	23.10 ^a,b^ ± 0.50	24.85 ^a^ ± 0.15	17.10 ^c^ ± 0.20	12.25 ^d^ ± 0.15	17.95 ^c^ ± 0.85	16.30 ^c^ ± 0.50	23.95 ^a^ ± 0.15	20.90 ^b^ ± 0.60
TSAAs (%)	2.83 ^c^ ± 0.12	3.48 ^b,c^ ± 0.02	3.31 ^b,c^ ± 0.05	3.90 ^b^ ± 0.06	2.92 ^c^ ± 0.17	5.59 ^a^ ± 0.31	3.37 ^b,c^ ± 0.15	3.29 ^b,c^ ± 0.06
Cys in TSAAs (%)	40.68 ^e,f^ ± 0.42	33.01 ^g^ ± 1.41	37.45 ^f,g^ ± 2.19	59.60 ^b^ ± 0.73	52.41 ^c^ ± 0.81	69.04 ^a^ ± 0.58	49.26 ^c,d^ ± 0.94	44.97 ^d,e^ ± 0.14
Total aromatic amino acids (TArAAs)	78.70 ^a^ ± 0.00	73.90 ^a^ ± 1.30	42.70 ^c^ ± 0.40	36.35 ^c^ ± 0.45	62.60 ^b^ ± 1.30	20.65 ^d^ ± 0.45	74.20 ^a^ ± 0.60	55.55 ^b^ ± 4.55
TArAAs (%)	9.63 ^b,c^ ± 0.21	10.35 ^a,b^ ± 0.18	8.28 ^c,d^ ± 0.04	11.59 ^a^ ± 0.19	10.18 ^a,b^ ± 0.09	7.09 ^d^ ± 0.33	10.44 ^a,b^ ± 0.49	8.77 ^b,c,d^ ± 0.62
P-PER	2.55 ^a^ ± 0.01	1.92 ^b^ ± 0.07	1.14 ^c,d^ ± 0.03	1.36 ^c^ ± 0.01	0.99 ^d^ ± 0.02	0.52 ^e^ ± 0.02	2.47 ^a^ ± 0.00	1.82 ^b^ ± 0.07
Leu/Ile ratio	1.82 ^c^ ± 0.02	1.91 ^c^ ± 0.08	1.09 ^d^ ± 0.02	4.09 ^a^ ± 0.09	1.24 ^d^ ± 0.04	3.32 ^b^ ± 0.15	1.81 ^c^ ± 0.00	1.94 ^c^ ± 0.04
Leu–Ile (difference)	33.60 ^a^ ± 0.20	28.85 ^a,b^ ± 1.95	3.25 ^d^ ± 0.55	32.85 ^a^ ± 0.45	7.35 ^d^ ± 1.05	16.30 ^c^ ± 0.70	32.35 ^a^ ± 0.15	26.90 ^b^ ± 0.90
Leu–Ile (difference %)	45.16 ^b^ ± 0.45	47.68 ^b^ ± 2.08	8.61 ^d^ ± 0.42	75.51 ^a^ ± 0.51	19.33 ^c^ ± 0.83	69.78 ^a^ ± 1.35	44.87 ^b^ ± 0.02	48.54 ^b^ ± 1.09
Major taste components								
Bitter AAs	254.50 ^a^ ± 0.70	214.90 ^c^ ± 0.20	173.25 ^f^ ± 1.15	111.95 ^g^ ± 0.15	179.50 ^e^ ± 0.00	77.55 ^h^ ± 0.45	250.55 ^b^ ± 0.35	194.00 ^d^ ± 0.10
Bitter AAs (%)	31.14 ^b,c^ ± 0.60	30.09 ^c,d^ ± 0.02	33.58 ^a,b^ ± 0.09	35.68 ^a^ ± 0.18	29.19 ^c,d^ ± 0.32	26.62 ^d^ ± 0.81	35.25 ^a^ ± 1.34	30.64 ^b,c^ ± 0.35
Savory (Umami) AAs	229.04 ^a^ ± 4.19	210.55 ^a,b^ ± 1.15	117.80 ^d,e^ ± 0.20	76.05 ^e,f^ ± 1.05	177.40 ^b,c^ ± 0.30	58.80 ^f^ ± 3.20	160.12 ^c^ ± 5.52	159.50 ^c,d^ ± 0.50
Savory (Umami) AAs (%)	27.99 ^a,b^ ± 0.92	29.48 ^a^ ± 0.17	22.83 ^c,d^ ± 0.13	24.24 ^b,c,d^ ± 0.25	28.86 ^a,b^ ± 0.37	20.21 ^d^ ± 1.59	22.43 ^c,d^ ± 1.44	25.19 ^a,b,c^ ± 0.36
Sweet AAs	143.55 ^a^ ± 5.15	138.75 ^a^ ± 0.45	117.45 ^a,b^ ± 0.15	57.95 ^c^ ± 0.45	132.55 ^a^ ± 0.15	91.20 ^b^ ± 9.50	124.68 ^a^ ± 4.09	138.25 ^a^ ± 2.85
Sweet AAs (%)	17.55 ^b^ ± 0.24	19.42 ^b^ ± 0.06	22.77 ^b^ ± 0.06	18.47 ^b^ ± 0.08	21.56 ^b^ ± 0.26	31.22 ^a^ ± 2.48	17.47 ^b^ ± 1.04	21.83 ^b^ ± 0.21

Values represent mean ± standard error. Means followed by different letters are significantly different (*p <* 0.05) according to Tukey’s *post-hoc* test.

**Table 7:** Amino acids scores and limiting amino acid of the selected medicinal plants’ parts following the FAO recommended (2013) amino acid scoring patterns for young children (6 months to 3 years).

Amino Acids	Studied Part with Medicinal Food Plant Applications				Studied Part Used Solely for Medicinal Applications			
	*A. cepa*	*S. aromaticum*	*T. tetraptera*	*Z. officinale*	*A. ringens*	*M. indica*	*P. biglobosa*	*P. nitida*
Histidine	1.41 ^a^ ± 0.00	1.12 ^b,c^ ± 0.03	0.94 ^d^ ± 0.03	0.31 ^f^ ± 0.01	1.04 ^c^ ± 0.02	0.45 ^e^ ± 0.01	1.17 ^b^ ± 0.02	0.91 ^d^ ± 0.02
Isoleucine	1.28 ^a^ ± 0.02	0.99 ^c^ ± 0.02	1.07 ^b^ ± 0.01	0.33 ^e^ ± 0.00	0.96 ^c^ ± 0.02	0.22 ^f^ ± 0.00	1.24 ^a^ ± 0.00	0.89 ^d^ ± 0.01
Leucine	1.13 ^a^ ± 0.00	0.92 ^b^ ± 0.02	0.57 ^e^ ± 0.01	0.66 ^d^ ± 0.00	0.58 ^e^ ± 0.01	0.35 ^f^ ± 0.01	1.09 ^a^ ± 0.00	0.84 ^c^ ± 0.01
Lysine	0.99 ^a^ ± 0.00	0.54 ^c^ ± 0.01	0.48 ^c^ ± 0.00	0.26 ^d,e^ ± 0.00	0.38 ^c,d^ ± 0.08	0.19 ^e^ ± 0.00	0.80 ^b^ ± 0.01	0.54 ^c^ ± 0.01
Methionine + cysteine	0.86 ^a,b^ ± 0.02	0.92 ^a^ ± 0.01	0.63 ^c^ ± 0.01	0.45 ^d^ ± 0.01	0.66 ^c^ ± 0.03	0.60 ^c^ ± 0.02	0.89 ^a^ ± 0.01	0.77 ^b^ ± 0.02
Phenylalanine + tyrosine	1.51 ^a^ ± 0.00	1.42 ^a^ ± 0.03	0.82 ^c^ ± 0.01	0.69 ^c^ ± 0.01	1.20 ^b^ ± 0.03	0.39 ^d^ ± 0.01	1.43 ^a^ ± 0.01	1.07 ^b^ ± 0.09
Threonine	1.12 ^a,b^ ± 0.15	0.99 ^a,b^ ± 0.03	0.81 ^b^ ± 0.03	0.74 ^b^ ± 0.01	0.93 ^b^ ± 0.02	1.69 ^a^ ± 0.30	0.89 ^b^ ± 0.14	0.87 ^b^ ± 0.03
Tryptophan	1.14 ^a,b^ ± 0.03	0.94 ^b,c^ ± 0.01	0.82 ^c,d^ ± 0.02	0.69 ^d,e^ ± 0.01	1.21 ^a^ ± 0.03	0.59 ^e^ ± 0.09	1.00 ^a,b,c^ ± 0.01	1.21 ^a^ ± 0.03
Valine	1.01 ^b^ ± 0.00	0.83 ^c,d^ ± 0.01	0.74 ^e^ ± 0.00	0.42 ^f^ ± 0.00	0.78 ^d^ ± 0.00	0.33 ^g^ ± 0.01	1.22 ^a^ ± 0.01	0.84 ^c^ ± 0.00
Most limiting AA	Methionine + cysteine	Lysine	Lysine	Lysine	Lysine	Lysine	Lysine	Lysine

Values represent mean ± standard error. Means followed by different letters are significantly different (*p <* 0.05) according to Tukey’s *post-hoc* test.

**Table 8:** Amino acids scores and limiting amino acid of the selected medicinal plants’ parts following the FAO recommended (2013) amino acid scoring patterns for older children, adolescents and adults.

Amino Acids	Studied Part with Medicinal Food Plant Applications				Studied Part Used Solely for Medicinal Applications			
	*A. cepa*	*S. aromaticum*	*T. tetraptera*	*Z. officinale*	*A. ringens*	*M. indica*	*P. biglobosa*	*P. nitida*
Histidine	1.76 ^a^ ± 0.00	1.39 ^b,c^ ± 0.04	1.17 ^d^ ± 0.03	0.39 ^f^ ± 0.01	1.29 ^c^ ± 0.02	0.57 ^e^ ± 0.01	1.46 ^b^ ± 0.02	1.14 ^d^ ± 0.02
Isoleucine	1.36 ^a^ ± 0.02	1.05 ^c^ ± 0.02	1.15 ^b^ ± 0.01	0.36 ^e^ ± 0.01	1.02 ^c^ ± 0.02	0.24 ^f^ ± 0.01	1.33 ^a^ ± 0.01	0.95 ^d^ ± 0.01
Leucine	1.22 ^a^ ± 0.00	0.99 ^b^ ± 0.02	0.62 ^e^ ± 0.01	0.71 ^d^ ± 0.00	0.62 ^e^ ± 0.01	0.38 ^f^ ± 0.01	1.18 ^a^ ± 0.00	0.91 ^c^ ± 0.01
Lysine	1.18 ^a^ ± 0.00	0.65 ^c^ ± 0.01	0.54 ^c^ ± 0.00	0.30 ^d,e^ ± 0.01	0.45 ^c,d^ ± 0.10	0.24 ^e^ ± 0.01	0.95 ^b^ ± 0.01	0.65 ^c^ ± 0.02
Methionine + cysteine	1.00 ^a,b^ ± 0.02	1.08 ^a^ ± 0.01	0.74 ^c^ ± 0.01	0.53 ^d^ ± 0.01	0.78 ^c^ ± 0.04	0.71 ^c^ ± 0.02	1.04 ^a^ ± 0.01	0.91 ^b^ ± 0.03
Phenylalanine + tyrosine	1.92 ^a^ ± 0.00	1.80 ^a^ ± 0.03	1.04 ^c^ ± 0.01	0.89 ^c^ ± 0.01	1.53 ^b^ ± 0.03	0.50 ^d^ ± 0.01	1.81 ^a^ ± 0.01	1.35 ^b^ ± 0.11
Threonine	1.39 ^a,b^ ± 0.19	1.23 ^a,b^ ± 0.03	1.01 ^b^ ± 0.03	0.92 ^b^ ± 0.01	1.15 ^b^ ± 0.02	2.10 ^a^ ± 0.37	1.11 ^b^ ± 0.18	1.08 ^b^ ± 0.03
Tryptophan	1.47 ^a,b^ ± 0.05	1.21 ^b,c^ ± 0.02	1.05 ^c,d^ ± 0.02	0.89 ^d,e^ ± 0.02	1.55 ^a^ ± 0.04	0.76 ^e^ ± 0.12	1.29 ^a,b,c^ ± 0.02	1.55 ^a^ ± 0.04
Valine	1.09 ^b^ ± 0.01	0.89 ^c,d^ ± 0.01	0.79 ^e^ ± 0.01	0.45 ^f^ ± 0.00	0.84 ^d^ ± 0.00	0.36 ^g^ ± 0.01	1.32 ^a^ ± 0.02	0.90 ^c^ ± 0.00
Most limiting AA	-	Lysine	Lysine	Lysine	Lysine	Lysine	Lysine	Lysine

Values represent mean ± standard error. Means followed by different letters are significantly different (*p <* 0.05) according to Tukey’s *post-hoc* test.

## Data Availability

The data presented in this study are available on request from the corresponding authors.

## References

[1] Abunnaja S.S., Sanchez J.A., Maulik N. (2013). Epidemiology of cardiovascular disease. Cardiovascular Diseases: Nutritional and Therapeutic Interventions.

[2] Lee C.H., Kim J.H. (2014). A review on the medicinal potentials of ginseng and ginsenosides on cardiovascular diseases. J. Ginseng Res..

[3] Kordalewska M., Markuszewski M.J. (2015). Metabolomics in cardiovascular diseases. J. Pharm. Biomed. Anal..

[4] Ozkan G., Kamiloglu S., Ozdal T., Boyacioglu D., Capanoglu E. (2016). Potential use of Turkish medicinal plants in the treatment of various diseases. Molecules.

[5] Grajeda-Iglesias C., Aviram M. (2018). Specific amino acids affect cardiovascular diseases and atherogenesis via protection against macrophage foam cell formation: Review article. Rambam Maimonides Med. J..

[6] WHO Cardiovascular Diseases. https://www.who.int/health-topics/cardiovascular-diseases/#tab=tab_1.

[7] Mota A.H. (2016). A review of medicinal plants used in therapy of cardiovascular diseases. Int. J. Pharmacogn. Phytochem. Res..

[8] Zhao C.N., Meng X., Li Y., Li S., Liu Q., Tang G.Y., Li H. (2017). Bin Fruits for prevention and treatment of cardiovascular diseases. Nutrients.

[9] Olorunnisola O.S., Bradley G., Afolayan A.J. (2011). Ethnobotanical information on plants used for the management of cardiovascular diseases in NKonkobe municipality, South Africa. J. Med. Plants Res..

[10] Di Paola R., Cordaro M., Crupi R., Siracusa R., Campolo M., Bruschetta G., Fusco R., Pugliatti P., Esposito E., Cuzzocrea S. (2016). Protective Effects of Ultramicronized palmitoylethanolamide (PEA-um) in myocardial ischaemia and reperfusion injury in vivo. Shock.

[11] Ssegawa P., Kasenene J.M. (2007). Medicinal plant diversity and uses in the Sango bay area, Southern Uganda. J. Ethnopharmacol..

[12] Ntie-Kang F., Lifongo L.L., Mbaze L.M.A., Ekwelle N., Owono Owono L.C., Megnassan E., Judson P.N., Sippl W., Efange S.M.N. (2013). Cameroonian medicinal plants: A bioactivity versus ethnobotanical survey and chemotaxonomic classification. BMC Complement. Altern. Med..

[13] Mounanga M.B., Mewono L., Angone S.A. (2015). Toxicity studies of medicinal plants used in sub-Saharan Africa. J. Ethnopharmacol..

[14] Eddouks M., Ajebli M., Hebi M. (2017). Ethnopharmacological survey of medicinal plants used in Daraa-Tafilalet region (Province of Errachidia), Morocco. J. Ethnopharmacol..

[15] Randriamiharisoa M.N., Kuhlman A.R., Jeannoda V., Rabarison H., Rakotoarivelo N., Randrianarivony T., Raktoarivony F., Randrianasolo A., Bussmann R.W. (2015). Medicinal plants sold in the markets of Antananarivo, Madagascar. J. Ethnobiol. Ethnomed..

[16] WHO Traditional, Complementary and Integrative Medicine. https://www.who.int/health-topics/traditional-complementary-and-integrative-medicine#tab=tab_1.

[17] Wu G. (2009). Amino acids: Metabolism, functions, and nutrition. Amino Acids.

[18] World Health Organization (2007). Protein and Amino Acid Requirements in Human Nutrition: Report of a Joint WHO/FAO/UNU Expert Consultation.

[19] Yang R.Y., Wang S.M., Sun L., Liu J.M., Li H.X., Sui X.F., Wang M., Xiu H.L., Wang S., He Q. (2015). Association of branched-chain amino acids with coronary artery disease: A matched-pair case-control study. Nutr. Metab. Cardiovasc. Dis..

[20] Ferguson J.F., Wang T.J. (2016). Branched-chain amino acids and cardiovascular disease: Does diet matter?. Clin. Chem..

[21] White P.J., Newgard C.B. (2019). Branched-chain amino acids in disease. Science.

[22] McDougall J. (2002). Plant foods have a complete amino acid composition. Circulation.

[23] Olsen T., Øvrebø B., Turner C., Bastani N.E., Refsum H., Vinknes K.J. (2018). Combining dietary sulfur amino acid restriction with polyunsaturated fatty acid intake in humans: A randomized controlled pilot trial. Nutrients.

[24] Ruiz-Canela M., Toledo E., Clish C.B., Hruby A., Liang L., Salas-Salvado J., Razquin C., Corella D., Estruch R., Ros E. (2016). Plasma branched-chain amino acids and incident cardiovascular disease in the PREDIMED Trial. Clin. Chem..

[25] Nie C., He T., Zhang W., Zhang G., Ma X. (2018). Branched chain amino acids: Beyond nutrition metabolism. Int. J. Mol. Sci..

[26] Tobias D.K., Lawler P.R., Harada P.H., Demler O.V., Ridker P.M., Manson J.A.E., Cheng S., Mora S. (2018). Circulating branched-chain amino acids and incident cardiovascular disease in a prospective cohort of US women. Circ. Genom. Precis. Med..

[27] Ntzouvani A., Nomikos T., Panagiotakos D., Fragopoulou E., Pitsavos C., McCann A., Ueland P.M., Antonopoulou S. (2017). Amino acid profile and metabolic syndrome in a male Mediterranean population: A cross-sectional study. Nutr. Metab. Cardiovasc. Dis..

[28] Xiao J., Bai W. (2019). Bioactive phytochemicals. Crit. Rev. Food Sci. Nutr..

[29] Odukoya J.O. (2015). Influence of Bioremediation on the Chemical and Nutritional Composition of Produce from Crude Oil Polluted Sites.

[30] Zhang Y.J., Gan R.Y., Li S., Zhou Y., Li A.N., Xu D.P., Li H.B., Kitts D.D. (2015). Antioxidant phytochemicals for the prevention and treatment of chronic diseases. Molecules.

[31] Geetha N., Harini K., Joseph M., Sangeetha R., Venkatachalam P. (2017). A comparison of microwave assisted medicinal plant extractions for detection of their phytocompounds through qualitative phytochemical and FTIR analyses. Iran. J. Sci. Technol. Trans. A Sci..

[32] Lillehoj H., Liu Y., Calsamiglia S., Fernandez-Miyakawa M.E., Chi F., Cravens R.L., Oh S., Gay C.G. (2018). Phytochemicals as antibiotic alternatives to promote growth and enhance host health. Vet. Res..

[33] Siyuan S., Tong L., Liu R.H. (2018). Corn phytochemicals and their health benefits. Food Sci. Hum. Wellness.

[34] Zhang L., Virgous C., Si H. (2019). Synergistic anti-inflammatory effects and mechanisms of combined phytochemicals. J. Nutr. Biochem..

[35] Patle T.K., Shrivas K., Kurrey R., Upadhyay S., Jangde R., Chauhan R. (2020). Phytochemical screening and determination of phenolics and flavonoids in *Dillenia pentagyna* using UV–vis and FTIR spectroscopy. Spectrochim. Acta Part A Mol. Biomol. Spectrosc..

[36] Bunaciu A.A., Aboul-Enein H.Y., Fleschin S. (2012). FTIR spectrophotometric methods used for antioxidant activity assay in medicinal plants. Appl. Spectrosc. Rev..

[37] da Silva Leite R., Hernandéz-Navarro S., do Nascimento M.N., Potosme N.M.R., Carrión-Prieto P., dos Santos Souza E. (2018). Nitrogen fertilization affects Fourier Transform Infrared spectra (FTIR) in *Physalis* L. species. Comput. Electron. Agric..

[38] Durak T., Depciuch J. (2020). Effect of plant sample preparation and measuring methods on ATR-FTIR spectra results. Environ. Exp. Bot..

[39] Johnson J., Mani J., Ashwath N., Naiker M. (2020). Potential for Fourier transform infrared (FTIR) spectroscopy toward predicting antioxidant and phenolic contents in powdered plant matrices. Spectrochim. Acta Part A Mol. Biomol. Spectrosc..

[40] Houston M.C. (2005). Nutraceuticals, vitamins, antioxidants, and minerals in the prevention and treatment of hypertension. Prog. Cardiovasc. Dis..

[41] Bleakley S., Hayes M. (2017). Algal proteins: Extraction, application, and challenges concerning production. Foods.

[42] Niittynen L., Nurminen M.-L., Korpela R., Vapaatalo H. (1999). Leena Niittynen’, Maria- Leena Nurminen2, Riitta Korpela’ and Heikki Vapaatalo2. Ann. Med..

[43] Lawin I.F., Laleye F.O.A., Agbani O.P., Assogbadjo A.E. (2015). Ethnobotanical assessment of the plant species used in the treatment of diabetes in the Sudano- Guinean zone of Benin. J. Anim. Plant Sci..

[44] Aumeeruddy M.Z., Mahomoodally M.F. (2020). Traditional herbal therapies for hypertension: A systematic review of global ethnobotanical field studies. S. Afr. J. Bot..

[45] Mahomoodally M.F., Protab K., Aumeeruddy M.Z. (2019). Medicinal plants brought by Indian indentured immigrants: A comparative review of ethnopharmacological uses between Mauritius and India. J. Ethnopharmacol..

[46] Mensah J.K., Okoli R.I., Turay A.A., Ogie-Odia E.A. (2009). Phytochemical analysis of medicinal plants used for the management of hypertension by Esan people of Edo State, Nigeria. Ethnobot. Leafl..

[47] Gbolade A. (2012). Ethnobotanical study of plants used in treating hypertension in Edo State of Nigeria. J. Ethnopharmacol..

[48] Olorunnisola O.S., Adetutu A., Afolayan A.J. (2014). An inventory of plants commonly used in the treatment of some disease conditions in Ogbomoso, South West, Nigeria. J. Ethnopharmacol..

[49] Yagi S.M., Yagi A.I. (2018). Traditional medicinal plants used for the treatment of diabetes in the Sudan: A review. Afr. J. Pharm. Pharmacol..

[50] Karou S.D., Tchacondo T., Djikpo Tchibozo M.A., Abdoul-Rahaman S., Anani K., Koudouvo K., Batawila K., Agbonon A., Simpore J., De Souza C. (2011). Ethnobotanical study of medicinal plants used in the management of diabetes mellitus and hypertension in the Central Region of Togo. Pharm. Biol..

[51] Odukoya J.O., Odukoya J.O., Ndinteh D.T. (2021). Elemental measurements and health risk assessment of sub-Saharan African medicinal plants used for cardiovascular diseases’ and related risk factors’ treatment. J. Trace Elem. Med. Biol..

[52] Nadembega P., Boussim J.I., Nikiema J.B., Poli F., Antognoni F. (2011). Medicinal plants in Baskoure, Kourittenga Province, Burkina Faso: An ethnobotanical study. J. Ethnopharmacol..

[53] Noumi E., Houngue F., Lontsi D. (1999). Traditional medicines in primary health care: Plants used for the treatment of hypertension in Bafia, Cameroon. Fitoterapia.

[54] Emmanuel M.M., Didier D.S. (2012). Traditional knowledge on medicinal plants use by Ethnic communities in Douala, Cameroon. Eur. J. Med. Plants.

[55] Kasali M.F., Mahano A.O., Bwironde F.M., Amani A.C., Mangambu J.D., Nyakabwa D.S., Wimba L.K., Tshibangu D.S.T., Ngbolua K.N., Kambale J.K. (2013). Ethnopharmacological survey of plant used against diabetes in Bukavu city (D. R. Congo). J. Ethnobiol. Tradit. Med..

[56] Katemo M., Mpiana P.T., Mbala B.M., Mihigo S.O., Ngbolua K.N., Tshibangu D.S.T., Koyange P.R. (2012). Ethnopharmacological survey of plants used against diabetes in Kisangani city (DR Congo). J. Ethnopharmacol..

[57] Demoz M., Gachoki K., Mungai K., Negusse B. (2015). Ethnobotanical survey and preliminary phytochemical studies of plants traditionally used for diabetes in Eritrea. Eur. J. Med. Plants.

[58] Bading Taika B., Bouckandou M., Souza A., Bourobou Bourobou H.P., MacKenzie L.S., Lione L. (2018). An overview of anti-diabetic plants used in Gabon: Pharmacology and toxicology. J. Ethnopharmacol..

[59] Mootoosamy A., Fawzi Mahomoodally M. (2013). Ethnomedicinal application of native remedies used against diabetes and related complications in Mauritius. J. Ethnopharmacol..

[60] Arowosegbe S., Olanipekun M.K., Kayode J. (2015). Ethnobotanical survey of medicinal plants used for the treatment of diabetes mellitus in Ekiti South Senatorial district, Nigeria. Eur. J. Bot. Plant Sci. Phytol..

[61] Oppong Bekoe E., Agyare C., Boakye Y.D., Baiden B.M., Asase A., Sarkodie J., Nettey H., Adu F., Otu P.B., Agyarkwa B. (2020). Ethnomedicinal survey and mutagenic studies of plants used in Accra metropolis, Ghana. J. Ethnopharmacol..

[62] Eleazu C.O., Awa K.C., Chukwuma E. (2012). Comparative study of the phytochemical composition of the leaves of five Nigerian medicinal plants. J. Biotechnol. Pharm. Res..

[63] Yemane B., Andebrhan M., Reddy K.S. (2017). Traditional medicinal plants used by Tigrigna ethnic group in Central Region of Eritrea. IOSR J. Pharm. Biol. Sci..

[64] De Smet P.A.G.M. (1998). Traditional pharmacology and medicine in Africa. Ethnopharmacological themes in sub-Saharan art objects and utensils. J. Ethnopharmacol..

[65] Jacques M.L., Xie Z., Xu X.J., Boping Y. (2015). Plants Used for the Treatment of Diabetes Mellitus in the Democratic Republic of Congo: Traditional Uses In Vitro and In Vivo. https://www.semanticscholar.org/paper/PLANTS-USED-FOR-THE-TREATMENT-OF-DIABETES-MELLITUS-Jacques-Xie/dd53a31e0fe4813fce5b9b08a4a636e82e510b17.

[66] Bekoe E., Kretchy I., Sarkodie J., Okraku A., Sasu C., Adjei D., Twumasi M. (2017). Ethnomedicinal survey of plants used for the management of hypertension sold in the Makola market, Accra, Ghana. Eur. J. Med. Plants.

[67] Diallo M.S.T., Traore M.S., Balde M.A., Camara A.K., Baldé E.S., Traore S., Oulare K., Diallo T.S., Laurent S., Muller R.N. (2019). Prevalence, management and ethnobotanical investigation of hypertension in two Guinean urban districts. J. Ethnopharmacol..

[68] Kamau L.N., Mbaabu M.P., Mbaria J.M., Karuri G.P., Kiama S.G. (2016). Knowledge and demand for medicinal plants used in the treatment and management of diabetes in Nyeri County, Kenya. J. Ethnopharmacol..

[69] Salihu Shinkafi T., Bello L., Wara Hassan S., Ali S. (2015). An ethnobotanical survey of antidiabetic plants used by Hausa-Fulani tribes in Sokoto, Northwest Nigeria. J. Ethnopharmacol..

[70] Kpodar M.S., Lawson-Evi P., Bakoma B., Eklu-Gadegbeku K., Agbonon A., Aklikokou K., Gbeassor M. (2015). Ethnopharmacological survey of plants used in the treatment of diabetes mellitus in south of Togo (Maritime Region). J. Herb. Med..

[71] Muyenga T.A., Musonda D., Chigunta M. (2018). Ethnobotanical survey of medical plants used in treatment of diabetes in Chipulukusu compound, Ndola district, Zambia. J. Prev. Rehabil. Med..

[72] Sabiu S., Madende M., Ayokun-nun Ajao A., Adepemi Ogundeji O., Lekena N., Adekunle Alayande K. (2019). The scope of phytotherapy in southern African antidiabetic healthcare. Trans. R. Soc. S. Afr..

[73] Ibrahim M.A., Habila J.D., Koorbanally N.A., Islam M.S. (2016). Butanol fraction of *Parkia biglobosa* (Jacq.) G. Don leaves enhance pancreatic β-cell functions, stimulates insulin secretion and ameliorates other type 2 diabetes-associated complications in rats. J. Ethnopharmacol..

[74] Tokoudagba J.M., Auger C., Bréant L., N’Gom S., Chabert P., Idris-Khodja N., Gbaguidi F., Gbenou J., Moudachirou M., Lobstein A. (2010). Procyanidin-rich fractions from *Parkia biglobosa* (Mimosaceae) leaves cause redox-sensitive endothelium-dependent relaxation involving NO and EDHF in porcine coronary artery. J. Ethnopharmacol..

[75] Yaoitcha A.S., Houehanou T.D., Fandohan A.B., Houinato M.R.B. (2015). Prioritization of useful medicinal tree species for conservation in Wari-Maro Forest Reserve in Benin: A multivariate analysis approach. For. Policy Econ..

[76] Konkon N., Ouatara D., Kpan W., Kouakou T. (2017). Medicinal plants used for treatment of diabetes by traditional practitioners in the markets of Abidjan district in Côte d’ Ivoire. J. Med. Plants Stud..

[77] Madingou N.O.K., Souza A., Lamidi M., Mengome L.E., Mba C.E.M., Bayissi B., Mavoungou I., Traore A.S. (2012). Study of medicinal plants used in the management of cardiovascular diseases at Libreville (Gabon): An ethnopharmacological approach. Int. J. Pharm. Sci. Res..

[78] Tjeck O.P., Souza A., Mickala P., Lepengue A.N., M’Batchi B. (2017). Bio-efficacy of medicinal plants used for the management of diabetes mellitus in Gabon: An ethnopharmacological approach. J. Intercult. Ethnopharmacol..

[79] Roldán E., Sánchez-Moreno C., de Ancos B., Cano M.P. (2008). Characterisation of onion (*Allium cepa* L.) by-products as food ingredients with antioxidant and antibrowning properties. Food Chem..

[80] Benitez V., Mollá E., Martín-Cabrejas A., López-Andréu F.J., Downes K., Terry L.A., Esteban R.M. (2011). Study of bioactive compound content in different onion sections. Plant Food Hum. Nutr..

[81] Liguori L., Califano R., Albanese D., Raimo F., Crescitelli A., Di Matteo M. (2017). Chemical composition and antioxidant properties of five white onion (*Allium cepa* L.) landraces. J. Food Qual..

[82] Pareek S., Sagar N.A., Sharma S., Kumar V. (2018). Onion (*Allium cepa* L.). Fruit Veg. Phytochem. Chem. Hum. Health.

[83] Marrelli M., Amodeo V., Statti G., Conforti F. (2019). Biological properties and bioactive components of *Allium cepa* L.: Focus on potential benefits in the treatment of obesity and related comorbidities. Molecules.

[84] Cortés-Rojas D.F., de Souza C.R.F., Oliveira W.P. (2014). Clove (*Syzygium aromaticum*): A precious spice. Asian Pac. J. Trop. Biomed..

[85] El-Maati M.F.A., Mahgoub S.A., Labib S.M., Al-Gaby A.M.A., Ramadan M.F. (2016). Phenolic extracts of clove (Syzygium aromaticum) with novel antioxidant and antibacterial activities. Eur. J. Integr. Med..

[86] Alfikri F.N., Pujiarti R., Wibisono M.G., Hardiyanto E.B. (2020). Yield, quality, and antioxidant activity of clove (*Syzygium aromaticum* L.) bud oil at the different phenological stages in young and mature trees. Scientifica.

[87] Batiha G.E.S., Alkazmi L.M., Wasef L.G., Beshbishy A.M., Nadwa E.H., Rashwan E.K. (2020). *Syzygium aromaticum* L. (Myrtaceae): Traditional uses, bioactive chemical constituents, pharmacological and toxicological activities. Biomolecules.

[88] Vicidomini C., Roviello V., Roviello G.N. (2021). Molecular basis of the therapeutical potential of clove. Molecules.

[89] Ngassoum M.B., Jirovetz L., Buchbauer G. (2001). SPME/GC/MS analysis of headspace aroma compounds of the Cameroonian fruit *Tetrapleura tetraptera* (Thonn.) Taub. Eur. Food Res. Technol..

[90] Aderibigbe A.O., Iwalewa E.O., Adesina S.K., Adebanjo A.O., Ukponmwan O.E. (2007). Anticonvulsant, analgesic and hypothermic effects of aridanin isolated from *Tetrapleura tetrapetra* fruit in mice. J. Biol. Sci..

[91] Kuate D., Kengne A.P.N., Biapa C.P.N., Azantsa B.G.K., Wan Muda W.A.M. (2015). Bin *Tetrapleura tetraptera* spice attenuates high-carbohydrate, high-fat diet-induced obese and type 2 diabetic rats with metabolic syndrome features. Lipids Health Dis..

[92] Adadi P., Kanwugu O.N. (2020). Potential application of *Tetrapleura tetraptera* and *Hibiscus sabdariffa* (Malvaceae) in designing highly flavoured and bioactive Pito with functional properties. Beverages.

[93] Mbaveng A.T., Chi G.F., Bonsou I.N., Abdelfatah S., Tamfu A.N., Yeboah E.M.O., Kuete V., Efferth T. (2020). *N*-acetylglycoside of oleanolic acid (aridanin) displays promising cytotoxicity towards human and animal cancer cells, inducing apoptotic, ferroptotic and necroptotic cell death. Phytomedicine.

[94] Saliu I.O., Amoo Z.A., Khan M.F., Olaleye M.T., Rema V., Akinmoladun A.C. (2021). Abatement of neurobehavioral and neurochemical dysfunctions in cerebral ischemia/reperfusion injury by *Tetrapleura tetraptera* fruit extract. J. Ethnopharmacol..

[95] Ahmad I., Zahin M., Aqil F., Hasan S., Khan M.S.A., Owais M. (2008). Bioactive compounds from *Punica granatum*, *Curcuma longa* and *Zingiber officinale* and their therapeutic potential. Drugs Future.

[96] Liu Y., Liu J., Zhang Y. (2019). Research progress on chemical constituents of *Zingiber officinale* Roscoe. Biomed. Res. Int..

[97] Mao Q.Q., Xu X.Y., Cao S.Y., Gan R.Y., Corke H., Beta T., Li H. (2019). Bin Bioactive compounds and bioactivities of ginger (*Zingiber officinale* Roscoe). Foods.

[98] Da Silveira Vasconcelos M., Mota E.F., Gomes-Rochette N.F., Nunes-Pinheiro D.C.S., Nabavi S.M., de Melo D.F., Nabavi S.M., Silva A.S. (2019). Ginger (*Zingiber officinale* Roscoe). Nonvitamin and Nonmineral Nutritional Supplements.

[99] Sulyman A.O., Akolade J.O., Sabiu S.A., Aladodo R.A., Muritala H.F. (2016). Antidiabetic potentials of ethanolic extract of *Aristolochia ringens* (Vahl.) roots. J. Ethnopharmacol..

[100] Ahmad J.B., Ajani E.O., Sabiu S. (2021). Chemical group profiling, in vitro and in silico evaluation of *Aristolochia ringens* on α-amylase and α-glucosidase activity. Evid. Based Complement. Altern. Med..

[101] Shah K., Patel M., Patel R., Parmar P. (2010). *Mangifera Indica* (Mango). Pharmacogn. Rev..

[102] Telang M., Dhulap S., Mandhare A., Hirwani R. (2013). Therapeutic and cosmetic applications of mangiferin: A patent review. Expert Opin. Ther. Pat..

[103] Ediriweera M.K., Tennekoon K.H., Samarakoon S.R. (2017). A review on ethnopharmacological applications, pharmacological activities, and bioactive compounds of *Mangifera indica* (Mango). Evid. Based Complement. Altern. Med..

[104] Alañón M.E., Oliver-Simancas R., Gómez-Caravaca A.M., Arráez-Román D., Segura-Carretero A. (2019). Evolution of bioactive compounds of three mango cultivars (*Mangifera indica* L.) at different maturation stages analyzed by HPLC-DAD-q-TOF-MS. Food Res. Int..

[105] Sellés A.J.N., Agüero J.A., Paz L.N. (2021). GC-MS analysis of mango stem bark extracts (*Mangifera indica* L.), Haden variety. Possible contribution of volatile compounds to its health effects. Open Chem..

[106] Komolafe K., Olaleye M.T., Fasan T.I., Elekofehinti O.O., Saliu J.A., Akindahunsi A.A. (2013). Lowering effect of *Parkia biglobosa* leaf saponins in Triton-X 1339-induced hyperlipidemic rats. Res. J. Pharm. Biol. Chem. Sci..

[107] Komolafe K., Olaleye T.M., Seeger R.L., Carvalho F.B., Boligon A.A., Athayde M.L., Klimaczewski C.V., Akindahunsi A.A., Rocha J.B.T. (2014). *Parkia biglobosa* improves mitochondrial functioning and protects against neurotoxic agents in rat brain hippocampal slices. Biomed. Res. Int..

[108] Alinde O.B.L., Esterhuyse A.J., Oguntibeju O.O. (2014). Potential role of *Parkia biglobosa* in the management and treatment of cardiovascular diseases. Antioxid. Antidiabetic Agents Hum. Health.

[109] Jauro S., Abubakar M.B., Geidam Y.A., Zanna M.Y., Kwoji I.D., Gulani I.A., Ibrahim I., Gharib H.S.A. (2018). Phytochemical and antimicrobial profile analysis of *Parkia biglobosa* against methicillin-resistant *Staphylococcus aureus*. J. Adv. Vet. Anim. Res..

[110] Menzies J.R.W., Paterson S.J., Duwiejua M., Corbett A.D. (1998). Opioid activity of alkaloids extracted from *Picralima nitida* (fam. Apocynaceae). Eur. J. Pharmacol..

[111] Erharuyi O., Falodun A., Langer P. (2014). Medicinal uses, phytochemistry and pharmacology of *Picralima nitida* (Apocynaceae) in tropical diseases: A review. Asian Pac. J. Trop. Med..

[112] Nazneen Bobby M.D., Wesely E.G., Johnson M. (2012). FT-IR studies on the leaves of *Albizia lebbeck* Benth. Int. J. Pharm. Pharm. Sci..

[113] Agatonovic-Kustrin S., Doyle E., Gegechkori V., Morton D.W. (2020). High-performance thin-layer chromatography linked with (bio)assays and FTIR-ATR spectroscopy as a method for discovery and quantification of bioactive components in native Australian plants. J. Pharm. Biomed. Anal..

[114] Adeyeye E.I., Akinyeye R.O., Ogunlade I., Olaofe O., Boluwade J.O. (2010). Effect of farm and industrial processing on the amino acid profile of cocoa beans. Food Chem..

[115] Salo-väänänen P.P., Koivistoinen P.E. (1996). Determination of protein in foods: Comparison of net protein and crude protein (N× 6.25) values. Food Chem..

[116] Mariotti F., Tomé D., Mirand P.P. (2008). Converting nitrogen into protein—Beyond 6.25 and Jones’ factors. Crit. Rev. Food Sci. Nutr..

[117] Adeyeye E.I. (2009). Amino acid composition of three species of Nigerian fish: *Clarias anguillaris*, *Oreochromis niloticus* and *Cynoglossus senegalensis*. Food Chem..

[118] Yust M.M., Pedroche J., Girón-Calle J., Vioque J., Millán F., Alaiz M. (2004). Determination of tryptophan by high-performance liquid chromatography of alkaline hydrolysates with spectrophotometric detection. Food Chem..

[119] Oriolowo O.B., John O.J., Mohammed U.B., Joshua D. (2020). Amino acids profile of catfish, crayfish and larva of edible dung beetle. Ife J. Sci..

[120] Sosulski F.W., Imafidon G.I. (1990). Amino acid composition and nitrogen-to-protein conversion factors for animal and plant foods. J. Agric. Food Chem..

[121] Marti-Quijal F.J., Zamuz S., Tomašević I., Gómez B., Rocchetti G., Lucini L., Remize F., Barba F.J., Lorenzo J.M. (2019). Influence of different sources of vegetable, whey and microalgae proteins on the physicochemical properties and amino acid profile of fresh pork sausages. LWT.

[122] Köhler R., Kariuki L., Lambert C., Biesalski H.K. (2019). Protein, amino acid and mineral composition of some edible insects from Thailand. J. Asia. Pac. Entomol..

[123] Tan X., Qi L., Fan F., Guo Z., Wang Z., Song W., Du M. (2018). Analysis of volatile compounds and nutritional properties of enzymatic hydrolysate of protein from cod bone. Food Chem..

[124] Kowalczewski P.Ł., Olejnik A., Białas W., Rybicka I., Zielińska-Dawidziak M., Siger A., Kubiak P., Lewandowicz G. (2019). The nutritional value and biological activity of concentrated protein fraction of potato juice. Nutrients.

[125] FAO (2013). Dietary Protein Quality Evaluation in Human Nutrition.

[126] FAO/WHO (1991). Protein Quality Evaluation.

[127] Mallappa R.H., Singh D.K., Rokana N., Pradhan D., Batish V.K., Grover S. (2019). Screening and selection of probiotic *Lactobacillus* strains of Indian gut origin based on assessment of desired probiotic attributes combined with principal component and heatmap analysis. LWT.

[128] Poojary M.M., Vishnumurthy K.A., Vasudeva Adhikari A. (2015). Extraction, characterization and biological studies of phytochemicals from *Mammea suriga*. J. Pharm. Anal..

[129] Teshika J.D., Zakariyyah A.M., Zaynab T., Zengin G., Rengasamy K.R., Pandian S.K., Fawzi M.M. (2018). Traditional and modern uses of onion bulb (*Allium cepa* L.): A systematic review. Crit. Rev. Food Sci. Nutr..

[130] Metrani R., Singh J., Acharya P., Jayaprakasha G.K., Patil B.S. (2020). Comparative metabolomics profiling of polyphenols, nutrients and antioxidant activities of two red onion (*Allium cepa* L.) cultivars. Plants.

[131] Odukoya J., Charles U., Odukoya J. (2019). Response of nutritional and phytochemical constituents of bitter leaf to some drying methods. Int. Res. J. Pure Appl. Chem..

[132] Adhikari B., Dhungana S.K., Waqas Ali M., Adhikari A., Kim I.D., Shin D.H. (2019). Antioxidant activities, polyphenol, flavonoid, and amino acid contents in peanut shell. J. Saudi Soc. Agric. Sci..

[133] Poggiogalle E., Fontana M., Giusti A.M., Pinto A., Iannucci G., Lenzi A., Donini L.M. (2019). Amino acids and hypertension in adults. Nutrients.

[134] Ntuli N.R. (2019). Nutrient content of scarcely known wild leafy vegetables from northern KwaZulu-Natal, South Africa. S. Afr. J. Bot..

[135] Turchini G.M., Hermon K.M., Francis D.S. (2018). Fatty acids and beyond: Fillet nutritional characterisation of rainbow trout (*Oncorhynchus mykiss*) fed different dietary oil sources. Aquaculture.

[136] Fredotović Ž., Soldo B., Šprung M., Marijanović Z., Jerković I., Puizina J. (2020). Comparison of organosulfur and amino acid composition between triploid onion *Allium cornutum* Clementi ex Visiani, 1842, and common onion *Allium cepa* L., and evidences for antiproliferative activity of their extracts. Plants.

[137] Neves D.A., Schmiele M., Pallone J.A.L., Orlando E.A., Risso E.M., Cunha E.C.E., Godoy H.T. (2019). Chemical and nutritional characterization of raw and hydrothermal processed jambu (*Acmella oleracea* (L.) R.K. Jansen). Food Res. Int..

[138] Esan Y.O., Omoba O.S., Enujiugha V.N. (2018). Biochemical and nutritional compositions of two accessions of *Amaranthus Cruentus* seed flour. Am. J. Food Sci. Technol..

[139] López D.N., Galante M., Robson M., Boeris V., Spelzini D. (2018). Amaranth, quinoa and chia protein isolates: Physicochemical and structural properties. Int. J. Biol. Macromol..

[140] Elharadallou S.B., Khalid I.I., Gobouri A.A., Abdel-Hafez S.H. (2015). Amino acid composition of cowpea (*Vigna ungiculata* L. Walp) flour and its protein isolates. Food Nutr. Sci..

[141] Sun C., Liu J., Yang N., Xu G. (2019). Egg quality and egg albumen property of domestic chicken, duck, goose, Turkey, quail, and pigeon. Poult. Sci..

[142] Luo D., Mu T.H., Sun H., Chen J. (2020). Optimization of the formula and processing of a sweet potato leaf powder-based beverage. Food Sci. Nutr..

[143] Parniakov O., Toepfl S., Barba F.J., Granato D., Zamuz S., Galvez F., Lorenzo J.M. (2018). Impact of the soy protein replacement by legumes and algae based proteins on the quality of chicken rotti. J. Food Sci. Technol..

[144] Jin H.J., Lee J.H., Kim D.H., Kim K.T., Lee G.W., Choi S.J., Chang P.S., Paik H.D. (2015). Antioxidative and nitric oxide scavenging activity of branched-chain amino acids. Food Sci. Biotechnol..

[145] Tobias D.K., Clish C., Mora S., Li J., Liang L., Hu F.B., Manson J.A.E., Zhang C. (2018). Dietary intakes and circulating concentrations of branched-chain amino acids in relation to incident type 2 diabetes risk among high-risk women with a history of gestational diabetes mellitus. Clin. Chem..

[146] Mendoza C. (2002). Effect of genetically modified low phytic acid plants on mineral absorption. Int. J. Food Sci. Technol..

[147] Lisiewska Z., Kmiecik W., Korus A. (2008). The amino acid composition of kale (*Brassica oleracea* L. var. acephala), fresh and after culinary and technological processing. Food Chem..

[148] Kaur N., Singh B., Kaur A., Yadav M.P., Singh N., Ahlawat A.K., Singh A.M. (2021). Effect of growing conditions on proximate, mineral, amino acid, phenolic composition and antioxidant properties of wheatgrass from different wheat (*Triticum aestivum* L.) varieties. Food Chem..

[149] Granato D., Santos J.S., Escher G.B., Ferreira B.L., Maggio R.M. (2018). Use of principal component analysis (PCA) and hierarchical cluster analysis (HCA) for multivariate association between bioactive compounds and functional properties in foods: A critical perspective. Trends Food Sci. Technol..

[150] Odukoya J.O., De Saeger S., De Boevre M., Adegoke G.O., Audenaert K., Croubels S., Antonissen G., Vermeulen K., Gbashi S., Njobeh P.B. (2021). Effect of selected cooking ingredients for nixtamalization on the reduction of *Fusarium* mycotoxins in maize and sorghum. Toxins.

